# Forward genetics reveals a conserved CNBHD-CNBHD interface that restrains EAG channel activation to regulate sleep

**DOI:** 10.1371/journal.pgen.1012255

**Published:** 2026-10-01

**Authors:** Xinyu Huang, Sudharsan Kannan, Gail A. Robertson, Han Wang

**Affiliations:** 1 Department of Biology, University of Wisconsin-Madison, Madison, Wisconsin, United States of America; 2 Genetics Training Program, University of Wisconsin-Madison, Madison, Wisconsin, United States of America; 3 Department of Neuroscience, School of Medicine and Public Health, University of Wisconsin-Madison, Madison, Wisconsin, United States of America; 4 Neuroscience Training Program, School of Medicine and Public Health, University of Wisconsin-Madison, Madison, Wisconsin, United States of America; University of California San Diego, UNITED STATES OF AMERICA

## Abstract

KCNH voltage-gated potassium channels regulate neuronal excitability, yet how their hallmark cyclic nucleotide-binding homology domains (CNBHDs) influence channel gating to regulate behavior remains unclear. Using an unbiased forward genetic screen in *Caenorhabditis elegans*, we identified a gain-of-function mutation in the EAG-subfamily KCNH channel EGL-2 that suppresses sleep. The mutation (G574E) alters a highly conserved glycine residue at the interface between CNBHDs of adjacent channel subunits. EGL-2 functions cell-autonomously in the central sleep-promoting neuron ALA. *In vivo* calcium imaging showed that the gain-of-function mutation suppresses sleep by inhibiting ALA neuronal activity, consistent with enhanced potassium channel activity. Genetic analysis demonstrated that the sleep-suppressing phenotype of G574E requires both an intact potassium selectivity filter and the intrinsic ligand-occupied CNBHD. A second substitution at the same glycine (G574R), which introduces a positively charged side chain in contrast to the negatively charged side chain in G574E, similarly suppresses sleep, indicating that perturbation of the CNBHD-CNBHD interface, rather than the specific amino acid change, underlies the phenotype. Moreover, mutations at the corresponding residue in the ERG-subfamily KCNH channels *C. elegans* UNC-103 and human hERG also produce gain-of-function effects, and electrophysiological analysis showed that hERG(G749R) shifts voltage-dependent channel activation to more negative potentials and accelerates activation kinetics. These findings identify EGL-2 as an important sleep regulator and uncover a conserved gating mechanism by which the intersubunit CNBHD-CNBHD interface restrains KCNH channel activation to regulate neuronal excitability and behavior.

## Author summary

KCNH voltage-gated potassium channels are key regulators of neuronal excitability, but how their gating is controlled to influence neural circuit activity and behavior remains unclear. Here, through forward genetics and functional analyses, we identify gain-of-function mutations in EGL-2, an EAG-subfamily KCNH potassium channel in *C. elegans*. These mutations suppress sleep by directly inhibiting a central sleep-promoting neuron and disrupt the intersubunit interface between adjacent cyclic nucleotide-binding homology domains (CNBHDs). Analogous mutations increase channel activity across multiple KCNH family members. These findings uncover an important role of EAG channels in sleep regulation and identify the intersubunit CNBHD-CNBHD interface as a previously unrecognized, conserved structural element that restrains KCNH channel activation.

## Introduction

Voltage-gated potassium channels are key determinants of neuronal excitability, shaping membrane potential dynamics and activity patterns of neural circuits that underlie behavior. Mutations in these channels can disrupt neuronal signaling and circuit function, leading to behavioral abnormalities. EAG (ether-à-go-go; Kv10) potassium channels belong to the KCNH or Kv10–12 family of voltage-gated potassium channels that also include ERG (ether-à-go-go-related gene; Kv11) and ELK (ether-à-go-go-like, Kv12), and they are widely expressed in the nervous system [1–3]. However, it remains incompletely understood how the gating properties of EAG channels are regulated to influence neural circuit activity and behavior.

KCNH channels share a conserved tetrameric structure with each subunit comprising an N-terminal Per-Arnt-Sim (PAS) domain, six transmembrane voltage-sensing and pore-forming segments (S1-S6), and a cyclic nucleotide-binding homology domain (CNBHD) linked to the pore via the C-linker [4–6]. Intersubunit PAS-CNBHD interactions form an intracellular gating ring [7]. Although structurally related to the cyclic nucleotide-binding domains of CNG and HCN channels [8–10], CNBHDs in KCNH channels do not bind cyclic nucleotides but instead bind a downstream “intrinsic ligand” that is important for function [11–13]. Cryo-electron microscopy (Cryo-EM) structures of rEag1 and hERG1a channels reveal that each CNBHD forms two intersubunit interfaces, one with a neighboring PAS domain and the other with a neighboring CNBHD [4,5]. While the PAS-CNBHD interactions have established roles in channel gating [6,12–15], it remains unclear whether the CNBHD-CNBHD interface contributes to channel gating.

Sleep is an evolutionarily conserved behavioral state that requires precisely regulated neuronal excitability within sleep-promoting circuits [16]. In *C. elegans*, cellular stress, such as noxious heat stress, can drive the animal into a sleep state, called stress-induced sleep (SIS), which is analogous to sickness sleep observed in other animals [17,18]. SIS in *C. elegans* is mediated by the conserved epidermal growth factor receptor (EGFR) signaling pathway primarily in the ALA neuron and, to a lesser extent, in the RIS neuron [19–21]. Upon activation of the EGFR signaling pathway, the ALA neuron is activated and releases a cocktail of neuropeptides that collectively drive the animal into SIS [22–24]. Unlike mammals, which have two EAG channels (EAG1 and EAG2), *C. elegans* has a single EAG channel, EGL-2. However, the role of this channel in sleep regulation is unknown.

Here, we performed a forward genetic screen for new regulators of sleep in *C. elegans* and identified gain-of-function mutations in *egl-2*. These mutations alter a highly conserved glycine located at the CNBHD-CNBHD interface. Using behavioral analysis, genetics, and calcium imaging, we show that the mutations reduce the excitability of the sleep-promoting neuron ALA, consistent with enhanced EGL-2/EAG channel activity. Equivalent mutations in *C. elegans* UNC-103/ERG and human hERG similarly enhance channel activity. Together, these findings identify EGL-2 as a key regulator of sleep in *C. elegans* and uncover a conserved molecular mechanism by which the CNBHD-CNBHD interface regulates KCNH channel gating and links channel function to neuronal activity and behavior.

## Results

### *egl-2(G574E)* mutants are defective in sleep

Transient overexpression of epidermal growth factor (EGF) robustly drives *C. elegans* into sleep, hereafter referred to as EGF‑induced sleep [19,21]. To identify genes important for EGF-induced sleep, we performed a forward genetic screen and isolated mutants that failed to sleep following conditional overexpression of EGF from a heat-shock-inducible transgene (Fig 1A). One of the strong sleep-defective mutants isolated in this screen was *sy985*, which mapped to the tip of the left arm of chromosome V. Several lines of evidence indicated that *egl-2/eag* is the causative gene underlying sleep defects of *sy985* mutants. First, whole-genome sequencing and Sanger sequencing of the genomic DNA from *sy985* mutants identified a G-to-A mutation in the coding sequence of *egl-2* (Fig 1B), resulting in a substitution of glycine 574 with glutamic acid (G574E) in the cyclic nucleotide-binding homology domain (CNBHD) of EGL-2 (Fig 2A). Second, genetic rescue with a fosmid containing wild-type *egl-2* genomic DNA sequence restored normal sleep in *egl-2(sy985)* mutants (Fig 1C). Third, *egl-2(tan200)*, a CRISPR-generated allele carrying the same mutation as *sy985*, displayed similar sleep defects (Fig 1D). For subsequent analysis, we used an outcrossed version of *egl-2(tan200)* to characterize the *egl-2(G574E)* mutation.

**Fig 1:**
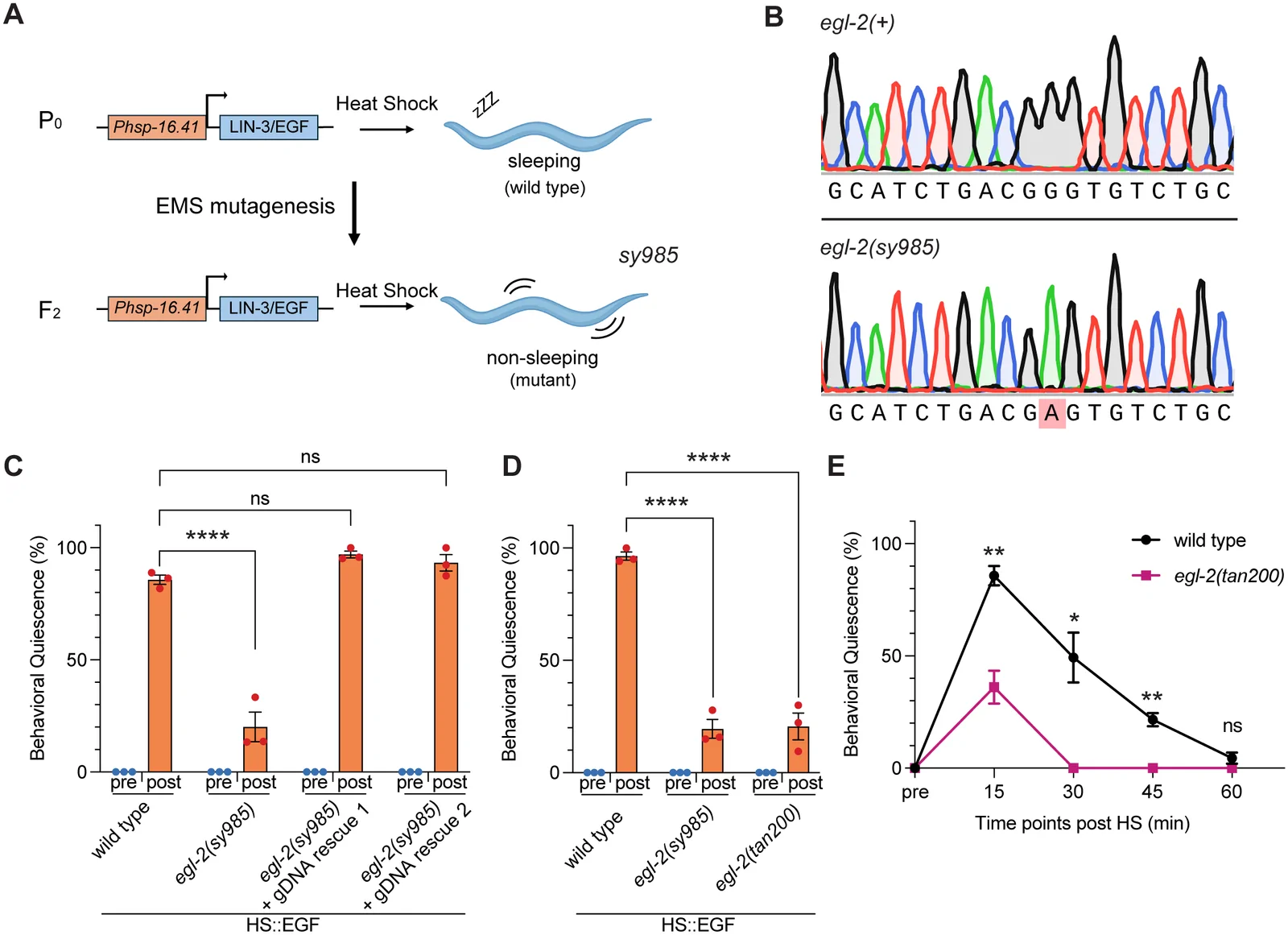
A forward genetic screen revealed that *egl-2(G574E)* mutants are defective in sleep. (**A**) Schematic of the forward genetic screen to isolate *egl-2(sy985)*. The parental strain (P_0_) contains the HS::EGF transgene, which drives worms into EGF-induced sleep after a brief heat shock. *Phsp-16.41* is a heat-shock-inducible promoter. F_2_ progeny of EMS-mutagenized parental animals were screened for sleep-defective mutants that exhibited feeding and locomotion after heat shock. The worm illustration was created in BioRender. Wang, H. (2026) https://BioRender.com/4ukdkrg. (**B**) Representative chromatograms from Sanger sequencing of the genomic DNA from *egl-2(+)* and *egl-2(sy985)* animals. A G-to-A point mutation (highlighted in the red box) was identified in the *egl-2* coding sequence of the *sy985* allele, resulting in a glycine-to-glutamic acid substitution at position 574 (G574E). (**C-D**) Quantification of sleep behavior as the fraction of quiescent worms for the indicated genotypes and conditions. All strains contain the transgene (HS::EGF) for conditional overexpression of EGF via 15-min heat shock (HS) at 33 °C to induce sleep. Pre and post refer to before and 2 h after heat shock, respectively. Each dot represents one independent assay containing 15–25 animals on a single plate. Mean ± SEM are shown. ns, not significant; ****, p < 0.0001. One-way ANOVA with Dunnett’s multiple-comparisons test for post-heat shock samples, comparing each genotype with wild type. (C) gDNA rescue 1 and 2 are two independent extrachromosomal arrays carrying the same fosmid containing the wild-type *egl-2* genomic sequence. (D) *egl-2(tan200)* is a CRISPR-generated allele carrying the same G574E substitution as *egl-2(sy985)*. (**E**) Time course of endogenous stress-induced sleep in animals of the indicated genotypes, shown as the fraction of quiescent worms at each time point. Pre refers to before heat shock. N2 and *egl-2(tan200)* animals were used as wild type and *egl-2(G574E)*, respectively. Data were collected from three independent replicates, with 15–25 animals of each strain assayed on separate plates in each replicate. Mean ± SEM are shown. ns, not significant; *, p < 0.05; **, p < 0.001. Two-tailed unpaired Student’s t-test for comparing wild type with *egl-2(tan200)* mutants at each time point.

**Fig 2:**
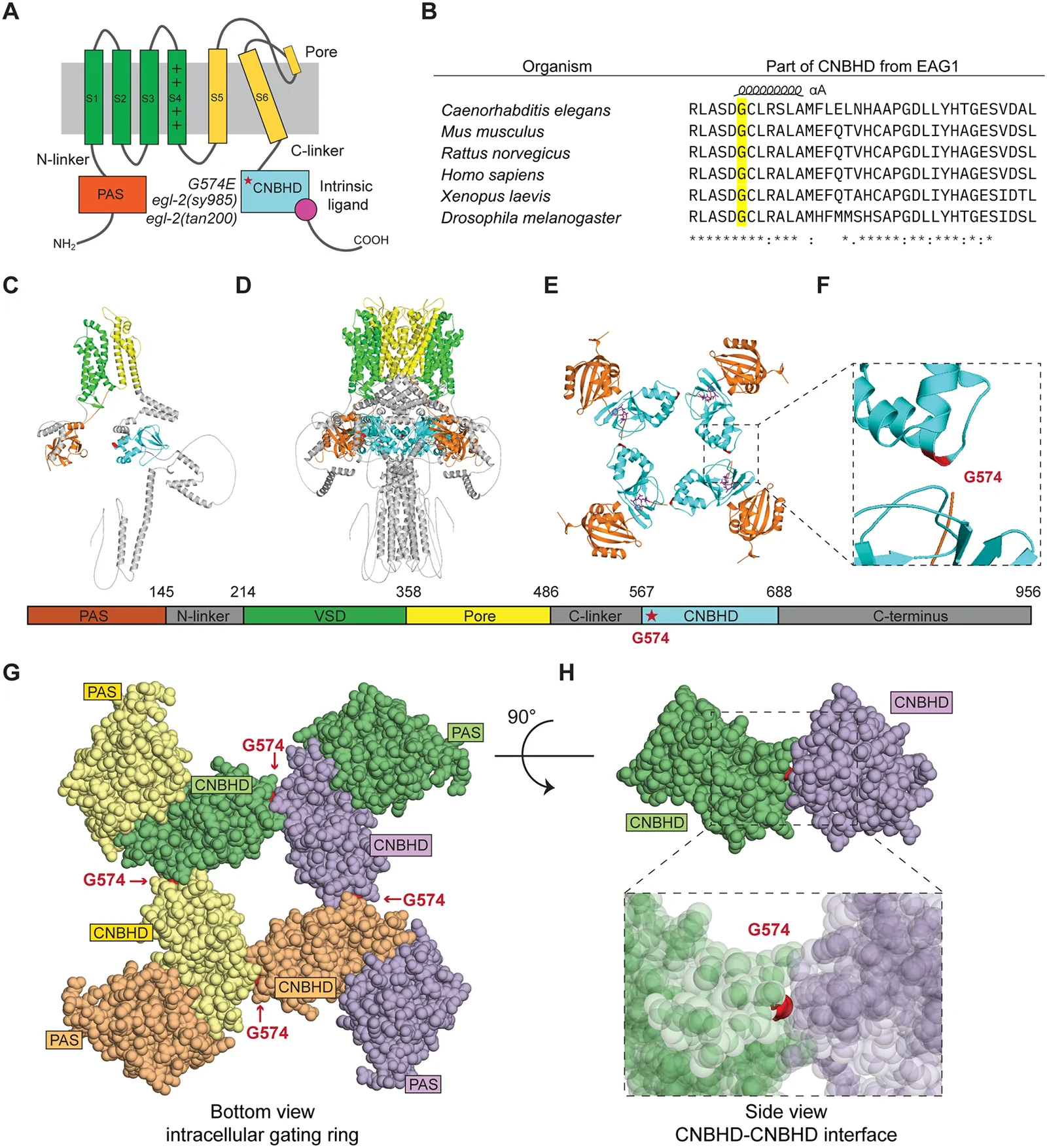
*egl-2* encodes the sole *C. elegans* EAG potassium channel. (**A**) Schematic of the domain organization and topology of the *C. elegans* EGL-2/EAG potassium channel. EGL-2 contains six transmembrane segments (S1-S6) and several intracellular domains, including an N-terminal PAS domain (orange) and a cyclic nucleotide-binding homology domain (CNBHD, cyan) with an intrinsic ligand (magenta). S1-S4 form a voltage sensor domain (green), whereas S5-S6 form a pore domain (yellow). The red star indicates the glycine residue (G574) mutated in *egl-2(sy985)* and *egl-2(tan200)*. Grey lines indicate the N-terminal region, interdomain linkers, and the C-terminus. The light gray rectangle indicates the plasma membrane. (**B**) Amino acid sequence alignment of a conserved region within the CNBHD of EAG1 orthologs from the indicated species. The glycine residue corresponding to G574 in EGL-2 (highlighted in yellow) is highly conserved and resides within the first α-helix of the CNBHD. Asterisks indicate identical residues; colons indicate conserved residues. (**C-F**) Structural models of EGL-2/EAG predicted by AlphaFold3, shown in cartoon representations, with the residue G574 highlighted in red. (C) Side view of a single subunit; (D) Side view of the tetrameric EGL-2 channel; (E) Bottom view of the tetrameric channel showing only the PAS domains and CNBHDs for better visualization of the location of the residue G574; (F) Magnified view of the region between two adjacent CNBHDs, as boxed in (E). Domains are colored as indicated in the diagram at the bottom, with residue numbering based on EGL-2 isoform a. The red star indicates the glycine (G574) mutated in *egl-2(sy985)* and *egl-2(tan200)*. (**G-H**) Sphere representations of the AlphaFold3-predicted structure of the homotetrameric EGL-2 channel, showing the intersubunit arrangement of the PAS domains and CNBHDs within the intracellular ring. The four subunits are colored yellow, green, orange, and purple. Other domains are omitted for better visualization of G574 (in red) at the CNBHD-CNBHD interface**.** (G) Bottom view of the intracellular gating ring of EGL-2 channel. The CNBHDs form the inner ring, with the PAS domains positioned peripherally. (H) Side view of two CNBHDs from adjacent subunits, highlighting the position of G574 (in red) at the interface, generated by a 90° downward rotation of the bottom views shown in (G) and displayed at higher magnification, with the PAS domains omitted for clarity. Top, opaque sphere representation of two adjacent CNBHDs; bottom, magnified view of a smaller region containing the CNBHD-CNBHD interface at 70% transparency.

Next, we tested whether *egl-2* is important for endogenous stress-induced sleep (SIS). To this end, we used a well-established heat-shock protocol [17] to induce SIS in animals without the conditional EGF overexpression transgene and compared the phenotypes of wild-type animals and *egl-2(G574E)* mutants. In this assay for endogenous SIS, wild-type worms enter a transient sleep state that peaks approximately 15 min after heat shock and return to wakefulness within about 60 min. By contrast, *egl-2(G574E)* mutants showed reduced behavioral quiescence along the time course after heat shock, indicating they are defective in endogenous SIS (Fig 1E). Taken together, our results demonstrate that *egl-2* is important for sleep regulation in *C. elegans*.

*egl-2* encodes the sole *C. elegans* homolog of EAG potassium channels, which are conserved across species (S1 Fig). The glycine residue mutated in *egl-2(G574E)* is in the first alpha helix of the CNBHD and is highly conserved (Fig 2A and 2B). Based on the cryo-EM structure of rEag1 [4] and the AlphaFold3-predicted structure of EGL-2 [25], the G574 residue lies at a conserved interface between two CNBHDs from adjacent subunits of EAG homotetrameric channels (Figs 2C-2H, S2, and S1 Video).

### *egl-2* functions in the ALA neuron to regulate sleep

*egl-2* has been reported to be expressed in neurons and muscles [26]. To determine the site of action of *egl-2* in sleep regulation, we first performed tissue-specific rescue experiments using single-copy transgenes. Neuron-specific expression of wild-type *egl-2* driven by a pan-neuronal promoter (*Pregf-1*) fully rescued the sleep defects of *egl-2(tan200)* mutants, whereas muscle-specific rescue of *egl-2* using a pan-muscle promoter (*Pmlc-2*) failed to correct the sleep defects (Fig 3A), indicating that *egl-2* functions in neurons to regulate sleep. As the ALA neuron and the RIS neuron are two major neurons in which the EGF receptor (EGFR) signaling is activated to induce SIS [19,20], we determined in which of these neurons *egl-2* functions to regulate sleep. We found that expression of wild-type *egl-2* specifically in the ALA neuron, but not the RIS neuron, restored normal EGF-induced sleep to *egl-2(tan200)* mutants (Fig 3A). Thus, *egl-2* functions in the ALA neuron to regulate sleep.

**Fig 3:**
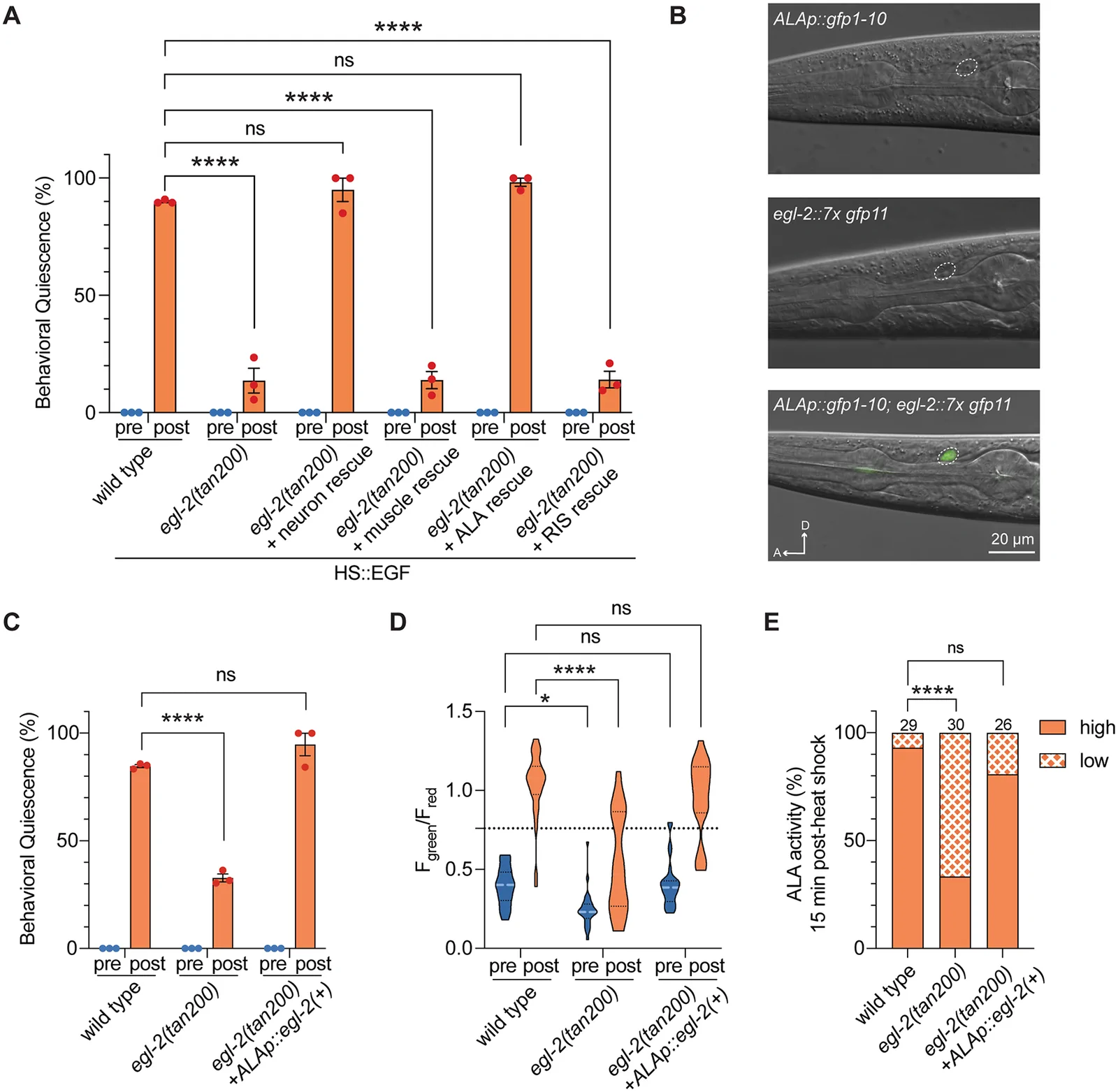
*egl-2* functions in the ALA neuron to regulate sleep. (**A**) Quantification of sleep behavior as the fraction of quiescent worms for the indicated genotypes and conditions. Tissue- and cell-specific rescue experiments in which wild-type *egl-2* cDNA was expressed specifically in neurons (*Prgef-1*), in muscles (*Pmlc-2*), in the ALA neuron (*Pnlp-67*), or in the RIS neuron (*Pflp-11*), respectively. All strains contain the transgene (HS::EGF) for conditional overexpression of EGF via 15-min heat shock (HS) at 33 °C to induce sleep. Pre and post refer to before and 2 h after heat shock, respectively. Each dot represents one independent assay containing 15–25 animals on a single plate. Mean ± SEM are shown. ns, not significant; ****, p < 0.0001. One-way ANOVA with Dunnett’s multiple-comparisons test for post-heat shock samples, comparing each genotype to wild type. (**B**) Representative images of the head region of L4 animals of the indicated genotypes showing EGL-2 expression in the ALA neuron using a split-GFP strategy. Top: *ALAp::gfp1–10* alone; middle: *egl-2::7x gfp11* alone; bottom: *ALAp::gfp1–10; egl-2::7x gfp11*. White dashed ovals outline the ALA neuron cell body. A, anterior; D, dorsal. Scale bar, 20 μm. (**C-D**) Quantification of endogenous stress-induced sleep (SIS) and ALA neuron activity in animals for the indicated genotypes and conditions. Pre and post refer to before and 15 min after heat shock, respectively. All strains contain the GCaMP6s transgene for calcium imaging. (C) SIS is quantified as the fraction of quiescent worms of the indicated genotypes and conditions. Each dot represents one independent assay containing 15–25 animals on a single plate. Mean ± SEM are shown. ns, not significant; ****, p < 0.0001. One-way ANOVA with Dunnett’s multiple-comparisons test for post-heat shock samples, comparing each genotype with wild type. (D) ALA neuron activity is quantified as the ratio of GCaMP6s to mCherry fluorescence measured in the cell body (F_green_/F_red_). Sample sizes n = 28, 29, 29, 30, 28, and 26, from left to right. The dotted line indicates the threshold (0.76) for high ALA activity, defined as the wild-type pre-heat shock mean + 3x standard deviation (SD), corresponding to the upper 99.7% bound of baseline activity. ns, not significant; *, p < 0.05; ****, p < 0.0001. Two-way ANOVA with Dunnett’s multiple-comparisons test for comparing each genotype with wild type under the same condition. (**E**) Fraction of animals with high or low ALA activity at 15 min post-heat shock. The same post-heat shock samples analyzed in (D) were categorized as having high or low activity based on the threshold defined in (D). Numbers above bars indicate the total animals analyzed. ns, not significant; ****, p < 0.0001. Two-sided Fisher’s exact test with Bonferroni correction for three pairwise comparisons.

Having shown that expression of *egl-2* in the ALA neuron is sufficient to rescue the sleep defects of *egl-2(tan200)* mutants, we next asked whether *egl-2* is endogenously expressed in the ALA neuron. A previously reported endogenously tagged *egl-2::yfp* strain [27] showed extremely dim fluorescence, making it difficult to determine the expression of EGL-2 in the ALA neuron. Thus, we used the NATF (Native and Tissue-Specific Fluorescence) strategy, a split-GFP approach that allows sensitive, cell-specific detection of endogenously tagged proteins by reconstituting two split GFP fragments (GFP1–10 and GFP11) into a functional fluorescent protein [28,29]. To do this, we used CRISPR/Cas9-mediated homology-directed repair to insert a *7x GFP11* tag into the endogenous *egl-2* locus, generating a strain expressing EGL-2 fused to seven tandem copies of GFP11 at its C-terminus. Neither this strain nor another strain carrying a single-copy transgene that expresses the complementary GFP1–10 fragment specifically in the ALA neuron showed detectable green fluorescence in the ALA neuron (Fig 3B). After crossing these two strains, we observed robust green fluorescence in the cell body of the ALA neuron (Fig 3B), indicating that EGL-2 is endogenously expressed in the ALA neuron. Taken together, these results demonstrate that *egl-2* functions cell-autonomously in the ALA neuron to regulate sleep.

### *egl-2(G574E*) suppresses the activity of the ALA neuron

During stress-induced sleep, the ALA neuron is activated and secretes several neuropeptides to promote sleep [22–24]. We therefore asked whether *egl-2*, which encodes an EAG potassium channel, regulates sleep by controlling ALA neuron activity. To this end, we generated a single-copy transgene (*ALAp::GCaMP6s::SL2::mCherry*) that simultaneously expresses the genetically encoded calcium indicator GCaMP6s [30] and the red fluorescent protein mCherry specifically in the ALA neuron. We used this strain to quantify ALA neuron activity 15 min after heat shock, corresponding to the peak of stress-induced sleep in wild-type animals, when nearly all wild-type animals were asleep, whereas *egl-2(G574E)* mutants exhibited severe sleep defects (Fig 1E).

Across all genotypes and conditions tested, ALA neuron activity closely paralleled the observed sleep phenotypes, indicating that the sleep defects of *egl-2(G574E)* are associated with impaired activation of this sleep-promoting neuron (Fig 3C and 3D). In the absence of heat shock, wild-type animals, *egl-2(G574E)* mutants, and the rescue line were awake and showed low ALA activity (Fig 3C and 3D). However, *egl-2(tan200)* mutants displayed a significantly lower GCaMP6s/mCherry ratio than wild type (Fig 3D), suggesting that the EGL-2(G574E) mutant channel suppresses basal ALA neuron activity. Following heat shock, ALA activity increased in both wild-type animals and *egl-2(G574E)* mutants, but the increase was markedly attenuated in *egl-2(G574E)* mutants (Fig 3D). Interestingly, *egl-2(tan200)* animals segregated into two distinct populations following heat shock, with 33% showing high ALA activity and 67% showing low ALA activity (Fig 3D and 3E). Strikingly, the fraction of *egl-2(tan200)* animals with low ALA activity matched the fraction of animals displaying sleep defects (Fig 3C and 3E). Expressing wild-type *egl-2* specifically in the ALA neuron rescued sleep and ALA activity defects of *egl-2(tan200)* mutants (Fig 3C-3E). Thus, these findings indicate that the *egl-2(G574E)* mutation suppresses sleep by reducing ALA neuron activity, consistent with decreased excitability of this sleep-promoting neuron.

### *egl-2(G574E)* is a gain-of-function allele

Because *egl-2(G574E*) suppresses ALA neuron activity, we hypothesized that G574E is a gain-of-function mutation that enhances EGL-2 potassium channel activity. We had multiple lines of additional evidence to support this hypothesis. First, as shown in Fig 4A-4C, *egl-2(G574E)* mutants exhibited similar sleep defects as the previously characterized gain-of-function allele *egl-2(n2656)* carrying an A478V mutation in the S6 transmembrane segment [26]; by contrast, *egl-2(tan232)* null mutants, in which the entire *egl-2* genomic DNA was replaced with a 43-bp STOP-IN cassette with multiple stop codons in all three reading frames [31], showed wild-type sleep. Second, we used CRISPR/Cas9 genome editing to knock out the mutated *egl-2(tan200[G574E])* gene by inserting the STOP-IN cassette into the third exon of the endogenous *egl-2(tan200)* locus (Fig 4A); we found that this genetic manipulation completely suppressed the sleep defects observed in *egl-2(tan200)* mutants (Fig 4D). Third, overexpression of *egl-2(G574E),* but not *egl-2 (+) cDNA,* specifically in the ALA neuron of otherwise wild-type animals was sufficient to cause sleep defects (Fig 4E), suggesting that *egl-2(G574E)* appears unlikely to cause the sleep defects simply by increasing the expression level of EGL-2.

**Fig 4:**
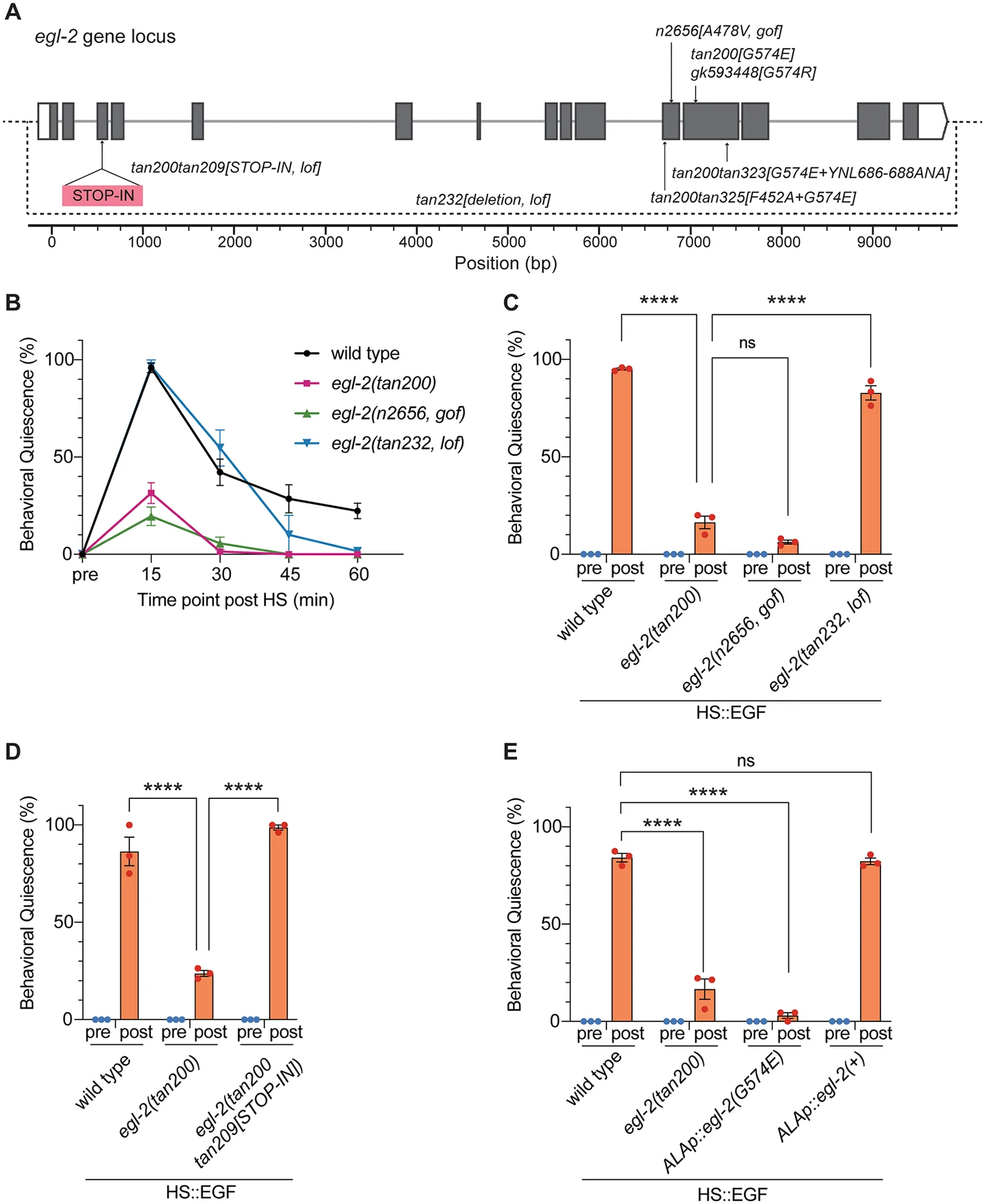
*egl-2(G574E)* is a gain-of-function allele. (**A**) Gene structure of *egl-2* showing the positions and the mutations of the indicated alleles. *gof*, gain-of-function; *lof*, loss-of-function. Open shapes, gray rectangles, and solid lines indicate UTRs, exons, and introns of *egl-2* isoform a, respectively. *egl-2(n2656[*A478V*])* is a well-characterized gain-of-function allele. *egl-2(gk593448)* carries a G574R mutation at the same glycine residue mutated in *egl-2(tan200[G574E])*. *egl-2(tan232)* is a loss-of-function allele with the entire *egl-2* gene deleted. STOP-IN is a 43-bp cassette that contains multiple stop codons in all three reading frames, which disrupts gene function when it is inserted into coding sequences [31]. *tan209*, *tan325*, and *tan323* were generated in the *egl-2(tan200)* mutant background using CRISPR. Some of these alleles were used in Figs 5 and 6. (**B**) Time course of endogenous stress-induced sleep in animals of the indicated genotypes, plotted as the fraction of quiescent worms at each time point. Pre refers to before heat shock. Data were from three independent replicates, with 15–25 animals of each strain assayed on separate plates in each replicate. Mean ± SEM are shown. (**C-E**) Quantification of sleep behavior shown as the fraction of quiescent worms for the indicated genotypes and conditions. All strains contain the transgene (HS::EGF) for conditional overexpression of EGF via 15-min heat shock (HS) at 33 °C to induce sleep. Pre and post refer to before and 2 h after heat shock, respectively. (C) The phenotype of *egl-2(tan200)* is similar to that of *egl-2(n2656, gof).* (D) *egl-2(tan200tan209[STOP-IN])* carries a STOP-IN cassette that disrupts the function of endogenous *egl-2(tan200[G574E])*. (E) *egl-2(G574E)* or *egl-2(+)* cDNA sequences were overexpressed in the ALA neuron of otherwise wild-type animals using an ALA-specific promoter (P*nlp-67*). Each dot represents one independent assay containing 15–25 animals on a single plate. Mean ± SEM are shown. ns, not significant; *, p < 0.05; ****, p < 0.0001. One-way ANOVA with Dunnett’s multiple-comparisons test for post-heat shock samples, comparing each genotype with *egl-2(tan200)* (C-D) or wild type (E).

To further determine whether the sleep defects observed in *egl-2(G574E)* mutants result from increased potassium ion efflux through the mutant channels, we disrupted ion permeation by mutating the potassium ion selectivity filter, which is highly conserved in all potassium channels (Fig 5A) [32,33]. It has been shown that the F456A mutation within the potassium selectivity filter of dEAG eliminates outward potassium currents [34]. Therefore, we used CRISPR/Cas9 genome editing to introduce the equivalent missense mutation, *egl-2(F452A),* into the endogenous *egl-2(G574E)* locus (Figs 4A and 5A). Remarkably, the *egl-2(F452A)* mutation completely suppressed the sleep defects observed in *egl-2(G574E)* mutants (Fig 5C), indicating that the sleep phenotype of *egl-2(G574E)* depends on the potassium ion selectivity filter. Taken together, these genetic results strongly indicate that EGL-2(G574E) is a gain-of-function mutation with increased potassium channel activity.

**Fig 5:**
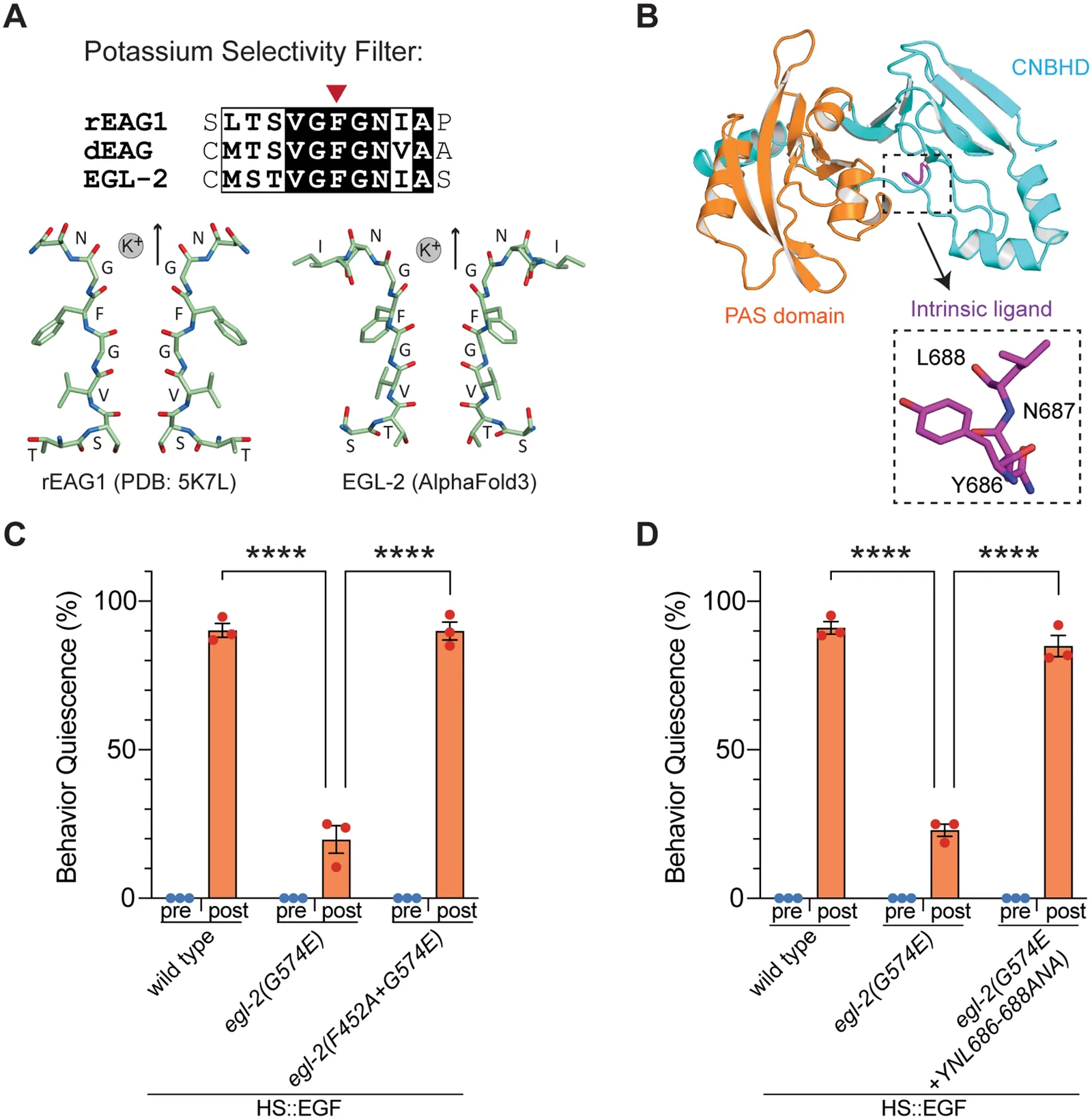
EGL-2(G574E) depends on the potassium selectivity filter and the intrinsic ligand. (**A**) Conservation of the potassium selectivity filter in EAG channels. Top panel: amino acid sequence alignment of the selectivity filter region of rEAG1, dEAG, and EGL-2. The red arrowhead marks the conserved phenylalanine (F) residue; its substitution with alanine (F456A) in dEAG abolishes potassium currents. Bottom panel: side views of the selectivity filter formed by two adjacent subunits of rEAG1 (PDB:5K7L) or EGL-2 (predicted by AlphaFold3). (**B**) Structural model of the PAS-CNBHD interaction in EGL-2, predicted by AlphaFold3. The PAS domain and CNBHD shown are from adjacent subunits. The intrinsic ligand motif (Y686N687L688), located near the end of CNBHD, is important for PAS-CNBHD interaction. (**C-D**) Quantification of sleep behavior shown as the fraction of quiescent worms for the indicated genotypes and conditions. All strains contain the transgene (HS::EGF) for conditional overexpression of EGF via 15-min heat shock (HS) at 33 °C to induce sleep. Pre and post refer to before and 2 h after heat shock, respectively. (C) EGL-2(F452A), the equivalent of the dEAG(F456A) mutation that abolishes potassium currents, was introduced into the endogenous *egl-2(tan200[G574E])* locus. (D) The intrinsic ligand motif (Y686N687L688) of EGL-2 was mutated to ANA in the endogenous *egl-2(tan200[G574E])* locus. Each dot represents one independent assay containing 15–25 animals on a single plate. Mean ± SEM are shown. ****, p < 0.0001. One-way ANOVA with Dunnett’s multiple-comparisons test for post-heat shock samples, comparing each genotype to *egl-2(G574E)* mutants.

### Gain-of-function EGL-2(G574E) depends on the intrinsic ligand of CNBHD

Because the G574 residue lies within the CNBHD, we determined whether the gain-of-function effect of EGL-2(G574E) requires a functional CNBHD. The cyclic nucleotide-binding pocket of the CNBHD in KCNH channels does not bind to cyclic nucleotides but instead is occupied by an intrinsic ligand located near the end of CNBHD (Fig 5B) [7,9]. The intrinsic ligand is important for normal CNBHD function [6]. In both EAG and hERG channels, intrinsic ligand mutations cause the same channel gating defects as deletion of the entire CNBHD [12,13]. For example, in human EAG1, mutating the intrinsic ligand (YNL to ANA) and deleting the CNBHD both produce a marked right shift in the conductance-voltage relationship and slow channel activation [12]. We therefore asked whether the self-liganded CNBHD is essential for the gain-of-function phenotype observed in *egl-2(G574E)* mutants*.* We used CRISPR-based genome editing to engineer the endogenous *egl-2(G574E)* locus by introducing the corresponding mutations in the intrinsic ligand (residues 686–688: YNL to ANA) (Figs 4A and 5B). We found that the YNL-to-ANA mutations completely suppressed the sleep defects of *egl-2(G574E)* mutants (Fig 5D). These results indicate that the gain-of-function effect of EGL-2(G574E) depends on its intact intrinsic ligand, suggesting that EGL-2(G574E) requires a functional CNBHD.

### *egl-2(G574R)* is also a gain-of-function allele

Our characterization of *egl-2(G574E)* suggested that disruption of the interface between two adjacent CNBHDs leads to increased channel activity. To further test this idea, we determined whether replacing the same glycine in EGL-2 with an arginine, a positively charged amino acid with a larger side chain and the opposite charge of glutamate, also produces a gain-of-function mutation. We found that *egl-2(G574R)* mutants displayed profound defects in both EGF-induced sleep and endogenous SIS (Fig 6A and 6C). In addition, similar to previously described gain-of-function *egl-2* mutants [26], both *egl-2(G574R)* and *egl-2(G574E)* mutants exhibited other behavioral phenotypes, including abnormal locomotion and defective egg laying. Notably, heterozygous *egl-2(G574R)* mutants showed more severe sleep defects than heterozygous *egl-2(G574E)* mutants (Fig 6B), and homozygous *egl-2(G574R)* mutants retained more eggs in the uterus than homozygous *egl-2(G574E)* mutants (S3 Fig)*.* Together, these results indicate that *egl-2(G574R)* is a stronger gain-of-function allele than *egl-2(G574E).*

**Fig 6:**
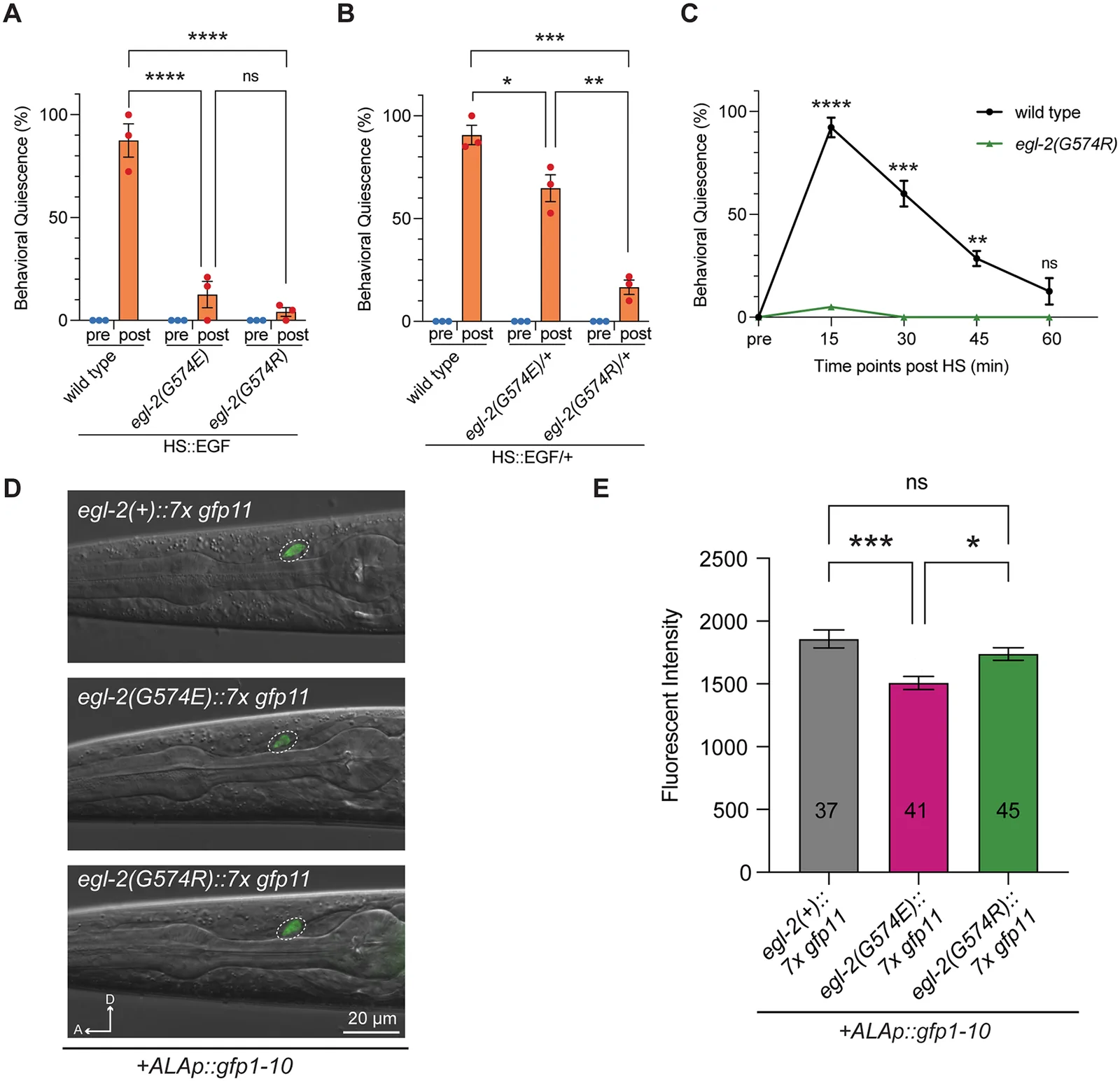
*egl-2(G574R)* is a gain-of-function allele and disrupts EGF-induced sleep. (**A-B**) Quantification of sleep behavior as the fraction of quiescent worms for the indicated genotypes and conditions. All strains contain the transgene (HS::EGF) for conditional overexpression of EGF via 15-min heat shock (HS) at 33 °C to induce sleep. Pre and post refer to before and 2 h after heat shock, respectively. *egl-2(tan200)* and *egl-2(gk593448)* were used as *egl-2(G574E)* and *egl-2(G574R*), respectively. Homozygous (A) and heterozygous (B) animals were assayed to compare the sleep phenotype. Each dot represents one independent assay containing 15–25 animals on a single plate. Mean ± SEM are shown. ns, not significant; *, p < 0.05; **, p < 0.01; ***, p < 0.001; ****, p < 0.0001. One-way ANOVA with Tukey’s multiple-comparisons test for post-heat shock samples. (**C**) Time course of endogenous stress-induced sleep in animals of the indicated genotypes shown as the fraction of quiescent worms at each time point. Pre refers to before heat shock. The *egl-2(gk583448)* allele was used as *egl-2(G574R)*. Data were collected from three independent replicates, with 15–25 animals of each strain assayed on separate plates in each replicate. Mean ± SEM are shown. ns, not significant; **, p < 0.01; ***, p < 0.001; ****, p < 0.0001. Two-tailed unpaired Student’s t-test for comparing wild type and *egl-2(gk583448)* mutants at each time point. (**D-E**) Representative images and quantification of endogenous EGL-2 protein expression in the ALA neuron using a split-GFP strategy. (D) Top: *egl-2(+)::7x gfp11*; Middle: *egl-2(G574E)::7x gfp11*; bottom: *egl-2(G574R)::7x gfp11* animals. All worm strains also carried the transgene *ALAp::gfp1–10* for ALA-specific expression of GFP1–10. White dashed ovals outline the ALA neuron cell body. A, anterior; D, dorsal. Scale bar, 20 μm. (E) Numbers shown on the bars indicate the total number of animals imaged for each genotype. ns, not significant; *, p < 0.05; ****, p < 0.0001. One-way ANOVA with Tukey’s multiple-comparisons test.

To rule out the possibility that G574E and G574R produce gain-of-function phenotypes by increasing EGL-2 expression, we again used the NATF strategy with split GFP [29] to quantify endogenous expression of EGL-2 in the ALA neuron. Compared to wild type, neither *egl-2(G574R)* nor *egl-2(G574E)* increased the expression level of EGL-2 in the ALA neuron; in fact, *egl-2(G574E)* had a slightly lower expression level (Fig 6D and 6E). Taken together, these results indicate the gain-of-function phenotypes of *egl-2(G574R)* and *egl-2(G574E)* likely arise from perturbation of the CNBHD-CNBHD interface rather than from the specific chemical properties of the substituted residue or increased expression of mutant channels, suggesting that the conserved interface is critical for regulating EGL-2 channel activity.

### *unc-103(G427R)* is a gain-of-function allele

EAG and ERG potassium channels share a similar overall tetrameric architecture that includes a conserved intersubunit interface between two CNBHDs from adjacent subunits (S2 Fig) [4,5], raising the possibility that the CNBHD-CNBHD interface may have a conserved role in regulating KCNH channel activity. Because the glycine residue at position 574 of EGL-2 is highly conserved among EAG and ERG channels (Fig 7A), we asked whether the corresponding mutation in ERG channels also increases their channel activity. We first tested this hypothesis in UNC-103, the sole *C. elegans* homolog of ERG channels and a key regulator for egg laying and male mating [35,36]. Because *egl-2(G574R)* is a stronger gain-of-function allele than *egl-2(G574E)* (Figs 6B and S3), we generated the corresponding *unc-103(G427R)* mutation using CRISPR/Cas9 genome editing and assessed its *in vivo* activity using a well-established egg-laying assay [37].

**Fig 7:**
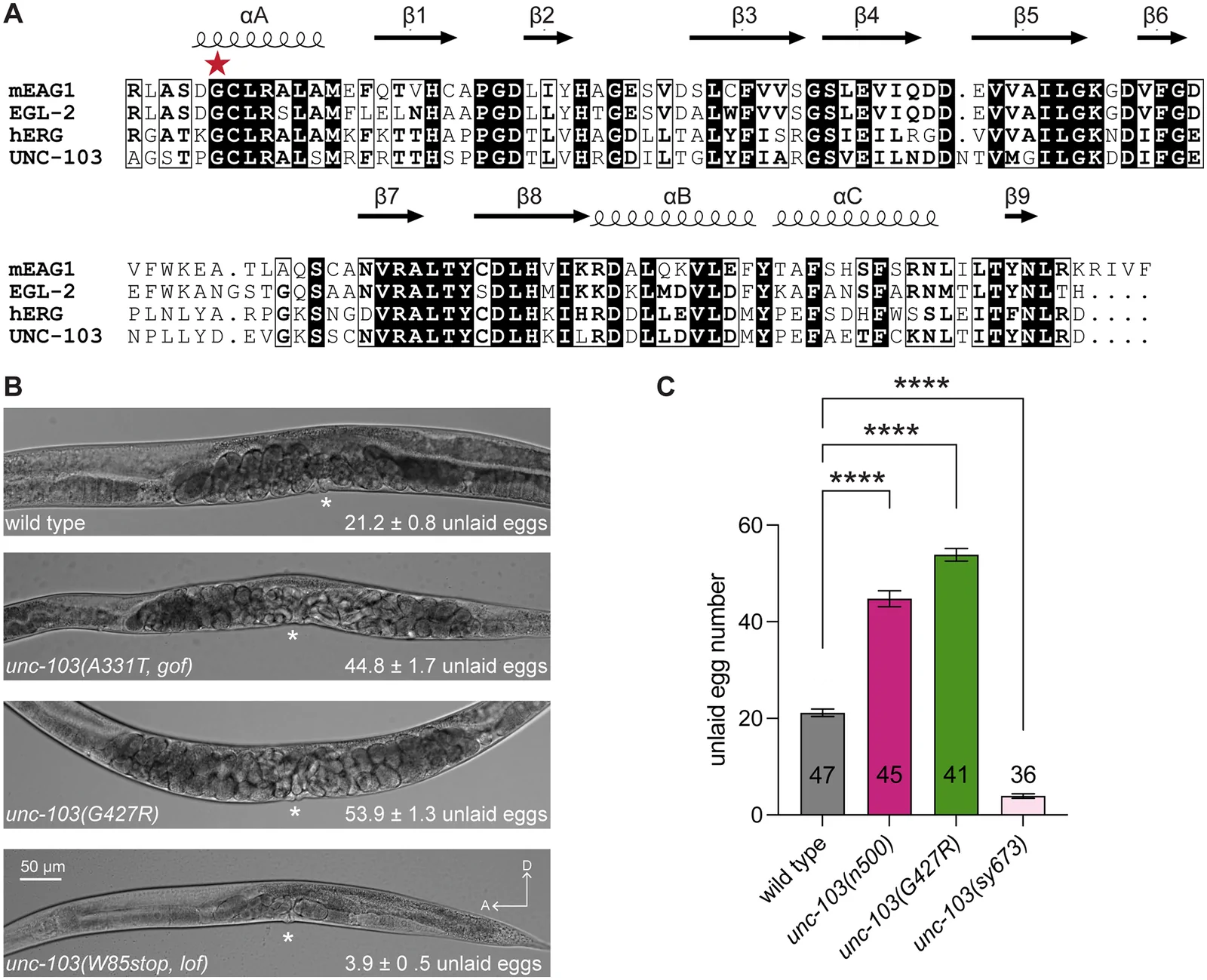
*unc-103*(G427R) is a gain-of-function allele that inhibits egg laying. (**A**) Amino acid sequence alignment of the CNBHDs from mEAG1, EGL-2, hERG, and UNC-103. Secondary structure elements from the mEAG1 CNBHD (PDB: 4F8A) are indicated above the alignment, with three α-helices (αA–αC) and nine β-strands (β1–β9) indicated by coils and arrows, respectively. The red star indicates the conserved glycine residue corresponding to mEAG1(G588), EGL-2(G574), UNC-103(G427), and hERG1a(G749). (**B-C**) Representative brightfield images and quantification of the eggs retained in the uterus of adult animals with the indicated genotypes. *gof*, gain-of-function; *lof*, loss-of-function. White asterisks mark the vulva position. (B) A, anterior; D, dorsal. Scale bar, 50 μm. (C) Numbers shown on the bars indicate the total number of animals scored for each genotype. Mean ± SEM are shown. ****, p < 0.0001. One-way ANOVA with Dunnett’s multiple-comparisons test for comparing each mutant genotype with wild type.

As previously reported [36], animals carrying the well-characterized gain-of-function allele *unc-103(A331T*) retained significantly more eggs in the uterus than wild type, whereas *unc-103(W85stop)* null mutants retained far fewer eggs (Fig 7B and 7C). Strikingly, *unc-103(G427R)* mutants exhibited marked egg retention comparable to that of *unc-103(A331T)* animals (Fig 7B and 7C). Thus, *unc-103(G427R)* is a gain-of-function mutation, indicating that the highly conserved glycine at the CNBHD-CNBHD interface also plays a conserved role in regulating ERG channel activity.

### G749R directly potentiates hERG1a gating

To directly determine how perturbation of the conserved glycine at the CNBHD-CNBHD interface affects channel gating, we introduced the corresponding glycine-to-arginine mutation (G749R) into hERG1a, a human KCNH family channel whose gating properties are well characterized and readily quantified using two-electrode voltage clamp recordings in *Xenopus laevis* oocytes.

Analysis of steady-state outward currents normalized to peak tail currents revealed enhanced outward currents over a broad range of voltages in hERG1a(G749R) channels relative to wild-type channels, consistent with a gain-of-function phenotype (Fig 8A-8C). Conductance-voltage relationships derived from normalized tail currents showed a marked hyperpolarizing shift in voltage dependence for hERG1a(G749R) mutant channels relative to hERG1a(WT) channels, with half-maximal activation voltage shifting from −23.97 ± 1.32 mV for hERG1a(WT) to −35.85 ± 1.23 mV for hERG1a(G749R) (Fig 8D and 8E). Thus, the increased current carried by mutant channels is explained, at least in part, by channel activation at more negative membrane potentials.

**Fig 8:**
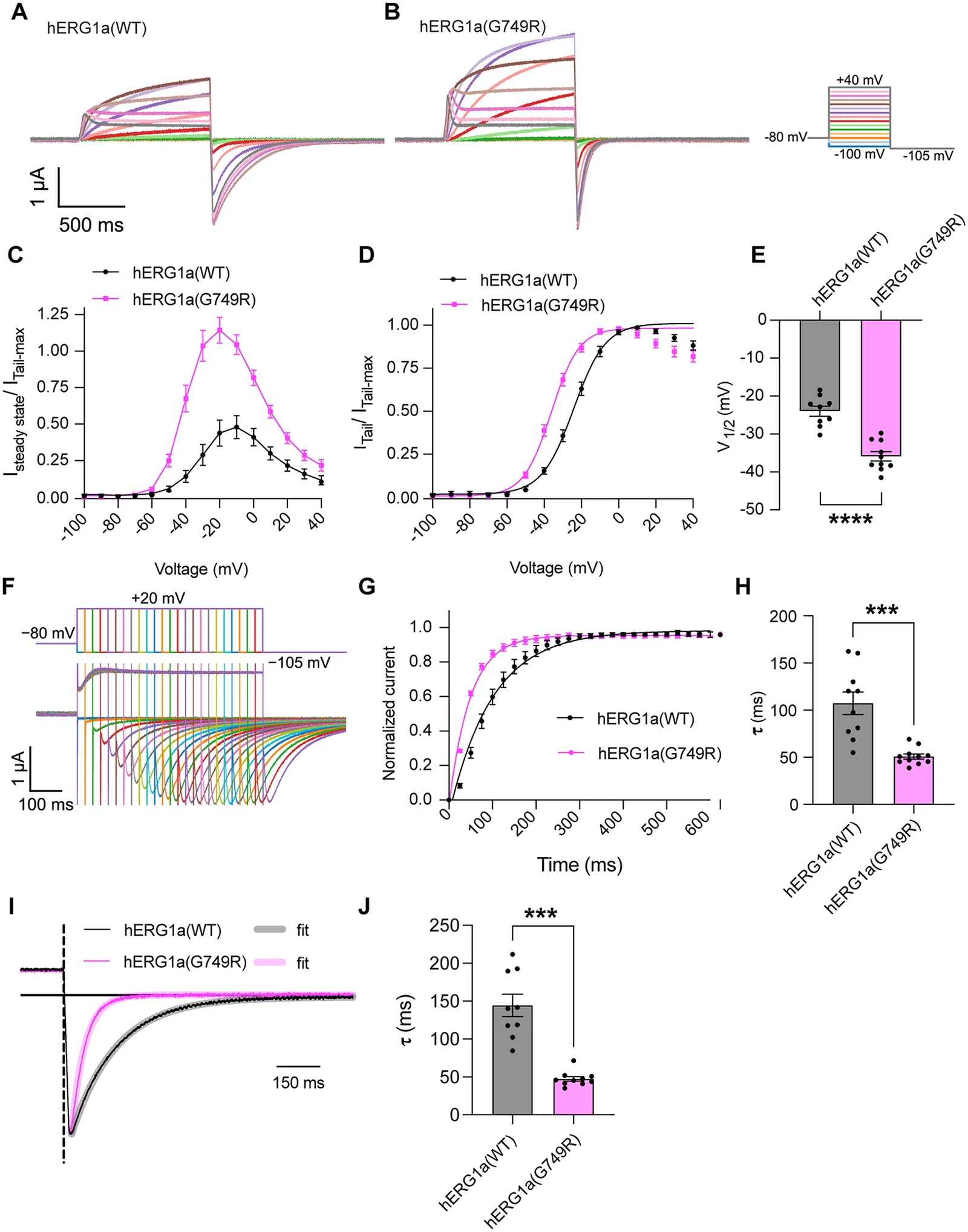
The G749R mutation produces gain-of-function gating changes in hERG channels assayed by two-electrode voltage clamp. (**A-B**) Representative current records from oocytes expressing (A) hERG1a(WT) and (B) hERG1a(G749R). From a holding potential of −80 mV, channels were depolarized to test potentials ranging from −100 to +40 mV in 10 mV increments, followed by a repolarizing step to −105 mV to record tail currents. (**C**) Plot of normalized steady-state current–voltage relationship for hERG1a(WT) and hERG1a(G749R) channels. Mean ± SEM are shown. (**D-E**) Voltage dependence of activation for hERG1a(WT) and hERG1a(G749R). (D) Plot of conductance-voltage relationship. Mean ± SEM are shown. Tail current amplitudes were normalized to the maximum tail current amplitude and plotted as a function of the preceding depolarization step to obtain the steady-state activation plots. The line of best fit is a Boltzmann function. hERG1a(WT), V_1/2_ = −23.97 mV, slope factor = 8.34; hERG1a(G749R), V_1/2_ = −35.85 mV, slope factor = 7.20. (E) Bar graph showing half-activation potential V_1/2_ derived from the Boltzmann fit of the conductance-voltage curves shown in (D). (**F**) Representative records of currents evoked using an envelope-of-tails protocol to generate the time course of activation. Channels were depolarized from a holding potential of −80 mV to +20 mV for progressively increasing durations in 25 ms increments, followed by repolarization to −105 mV to record tail currents. (**G-H**) Activation kinetics of hERG1a(WT) and hERG1a(G749R). (G) Time course of activation was constructed using the envelope‑of‑tails protocol illustrated in (F). The normalized peak tail current was plotted as a function of pulse duration and fitted with a single‑exponential function to determine the activation time constant. Mean ± SEM are shown. (H) Bar graph showing activation time constant derived from single-exponential fit to the time courses of activation shown in (G*)*. (**I-J**) Deactivation kinetics of hERG1a(WT) and hERG1a(G749R). (I) Representative traces of tail currents of hERG1a(WT) and hERG1a(G749R). Tail currents recorded at −105 mV with a + 40 mV prepulse are shown. Currents were normalized to their peak current amplitude to compare deactivation kinetics. Single exponential fits are superimposed using wider, lighter lines that match the data colors. (J) Bar graph showing deactivation time constant derived from single-exponential fit to deactivating currents. For (E, H, and J), each dot represents an independent oocyte. Mean ± SEM are shown. *** indicates p < 0.001, **** indicates p < 0.0001. Unpaired t-test with Welch’s correction.

To determine whether the G749R mutation also altered activation kinetics, we used an envelope-of-tails protocol to measure the time course of activation independent of the faster inactivation process of hERG (Fig 8F) [38]. Peak tail currents were plotted as a function of depolarization duration and fitted with a single-exponential function (Fig 8G and 8H). hERG1a(G749R) channels activated approximately twice as fast as hERG1a(WT) channels, with activation time constants decreasing from 107.27 ± 11.85 ms to 50.87 ± 2.72 ms.

We next examined deactivation kinetics using tail currents recorded at −105 mV following activation at +40 mV (Fig 8I). Single-exponential fits revealed that hERG1a(G749R) channels deactivated significantly faster than hERG1a(WT) channels, with deactivation time constants decreasing from 144.41 ± 14.75 ms to 47.44 ± 3.09 ms (Fig 8J).

Together, these findings demonstrate that the G749R mutation enhances hERG1a channel function by shifting activation to more negative voltages and accelerating channel opening. The accompanying acceleration of deactivation indicates that the mutation does not simply stabilize the open state. Rather, the data are consistent with the idea that interactions between adjacent CNBHDs normally constrain gating transitions and that disrupting this interface lowers one or more energetic barriers separating conformational states in the hERG gating pathway.

## Discussion

In this study, we combined forward genetics, behavioral analysis, *in vivo* calcium imaging, genome editing, and electrophysiology to uncover a previously unrecognized mechanism regulating KCNH potassium channel function. An unbiased screen for sleep-defective mutants in *C. elegans* identified gain-of-function mutations in the EAG potassium channel EGL-2 that alter a highly conserved glycine residue located at the interface between CNBHDs of adjacent subunits. We found that mutant EGL-2 channels disrupt sleep by suppressing the activity of the sleep-promoting neuron ALA, and that the sleep phenotype depends on potassium ion conduction and an intact intrinsic ligand within the CNBHD, consistent with increased channel activity. Introduction of the corresponding mutation into the *C. elegans* ERG channel UNC-103 also results in a gain-of-function allele, indicating that the role of this conserved residue extends across multiple KCNH family members. Importantly, direct measurements of hERG channel activity revealed that perturbation of the CNBHD-CNBHD interface by mutating the equivalent glycine residue directly promotes channel activation, providing mechanistic insight into how this conserved interface regulates KCNH channel function. Together, our findings identify EGL-2 as a new regulator of sleep and establish the intersubunit CNBHD-CNBHD interface as a conserved structural determinant of KCNH channel gating.

### EAG channels and sleep regulation

Our findings of EGL-2 as a new sleep regulator in *C. elegans* build on prior work describing roles for other voltage-gated potassium channels in promoting sleep. In *Drosophila*, loss of the Shaker potassium channel dramatically reduces sleep [39]. In *C. elegans*, deletion of the BK channel SLO-1 shortens sleep duration [40]. Similarly, depletion of the BK channel KCav1.1 reduces slow-wave sleep in mice [41]. In contrast to these examples, our study identifies gain-of-function mutations in EGL-2, an EAG channel, that act in the sleep-promoting neuron ALA to suppress sleep. Several observations support the conclusion that the sleep phenotype results from enhanced potassium channel function rather than altered channel expression. First, the mutant phenotype requires an intact potassium selectivity filter. Second, overexpression of the wild-type *egl-2* in the ALA neuron does not cause sleep defects. Third, both *egl-2(G574E)* and *egl-2(G574R)* suppress sleep without increasing EGL-2 channel expression in the ALA neuron. Fourth, the activity of the ALA neuron is inhibited in *egl-2(G574E)* mutants. Together, these findings support a model in which increased EGL-2 activity reduces the excitability of the ALA neuron, thereby suppressing sleep. In this regard, EGL-2 appears to act as a brake on activation of the ALA neuron, with gain-of-function mutations preventing the sustained neuronal activation thought to be required for ALA to release sleep-promoting neuropeptides and induce sleep. More broadly, our results demonstrate how increased potassium channel activity can reshape a behavioral state by altering the excitability of a single sleep-promoting neuron.

Our findings may also have implications beyond *C. elegans*. EAG1 and EAG2 are broadly expressed throughout the mammalian nervous system [42], and sleep disturbances have been reported in patients carrying gain-of-function EAG1 variants, including KCNH1(R357Q) [43]. Although the neural circuits involved are unknown, the conservation of both EAG channel structure and gain-of-function phenotypes raises the possibility that EAG channels contribute to sleep regulation across species.

Interestingly, although *egl-2(G574E)* is a gain-of-function allele, expression of wild-type *egl-2* rescued the sleep defects of *egl-2(G574E)* mutants (Fig 1C). Given that EGL-2 channel is a tetramer, overexpression of the wild-type EGL-2 subunit from the rescue transgenes likely dilutes the mutant EGL-2(G574E) subunit during channel assembly, dramatically reducing the fraction of fully assembled EGL-2(G574E) channels and thus rescuing the sleep defects. It is also worth noting that no obvious abnormal sleep phenotypes were observed in *egl-2* loss-of-function alleles in our assays (Fig 4B-4D), consistent with previous observations that animals lacking *egl-2* have grossly normal behavioral phenotypes [26]. We speculate that, in wild type, EGL-2 functions redundantly with other potassium channels to control proper excitability of the ALA neuron. Indeed, the *C. elegans* genome encodes about 70 potassium channel genes, many of which are expressed in the ALA neuron, as detected by single-cell RNA-seq [23,44,45]. The lack of abnormal sleep phenotypes in *egl-2* loss-of-function mutants may reflect functional compensation by other potassium channels expressed in the ALA neuron.

### A new class of KCNH gain-of-function mutations at the CNBHD-CNBHD interface

Our work identifies a previously unrecognized class of gain-of-function mutations in KCNH channels. Most previously characterized gain-of-function mutations of EAG and ERG channels are located within the voltage-sensing domain, the channel pore, or the C-linker [26,46–49]. For example, the classic gain-of-function mutations EGL-2(A478V) and UNC-103(A331T), as well as the corresponding mutation hERG(A653T) and others, occur within the transmembrane S6 segment and enhance channel activity [26,46,48]. In contrast, the mutations described here alter a highly conserved glycine located within the first α-helix of the CNBHD at the interface between adjacent CNBHDs. Notably, this interface lies opposite the extensively studied intersubunit PAS-CNBHD interface that is well known to regulate KCNH channel gating [6,12–15].

Several observations suggest that the gain-of-function phenotype does not arise from general disruption of the CNBHD. In mammalian KCNH channels, deletion of the CNBHD or mutations disrupting the intrinsic ligand or CNBHD-PAS interactions reduce channel activity [12,13,50,51], a phenotype distinct from those observed here. Moreover, many disease-associated mutations within the CNBHD impair channel function or trafficking and are therefore considered loss-of-function [15,51,52]. The dependence of EGL-2(G574E) on the intact intrinsic ligand of CNBHD further argues that the mutant channel retains a functional CNBHD. Without structures of the mutated EGL-2 channels, our study cannot completely rule out the possibility that the EGL-2(G574E) and EGL-2(G574R) mutations cause gain-of-function effects by altering individual CNBHD dynamics or CNBHD-to-pore communication. However, we favor the interpretation that both mutations cause gain-of-function effects by disrupting the CNBHD-CNBHD interface, because G574 sits at the interface and both substitutions, despite introducing oppositely charged side chains, produce similar phenotypes. As the corresponding glycine residue is similarly positioned at the interface in mammalian EAG and ERG tetramer channels [4,5], our findings support the conclusion that the conserved CNBHD-CNBHD interface plays an active role in regulating KCNH channel gating.

### A pre-open state model for CNBHD-CNBHD-mediated gating

How do mutations at the CNBHD-CNBHD interface increase channel activity? Our electrophysiological analysis of hERG1a suggests that these mutations alter the energetics of channel activation rather than simply destabilizing the closed state or disrupting the CNBHD. The G749R mutation shifted activation to more negative voltages and accelerated activation kinetics, indicating that mutant channels require less depolarization and less time to reach the open state. Deactivation was also accelerated, demonstrating that the mutation affects both forward and reverse gating transitions. One possible interpretation of these findings is that the mutation stabilizes an activation-permissive conformation and also lowers one or more energetic barriers separating closed and open states.

Evidence from several channels spanning all three KCNH subfamilies (EAG, ERG, and ELK) supports the existence of such activation-permissive intermediate states that lie closer to the open state than the resting state. In EAG channels, hyperpolarizing prepulses delay activation, evoking a nonsuperimposable Cole-Moore shift thought to arise by driving channels into remote closed states [53–55]. Conversely, depolarizing prepulses accelerate activation, presumably by initiating activation from closed states that lie closer to the open state [54,55]. Similarly, ELK and hERG channels also exhibit prepulse-dependent shifts in the voltage-dependence of activation [11,56–58]. Recent studies have identified intermediate nonconducting states in EAG channels that may represent activation-permissive conformations along the gating pathway [59–61].

We therefore propose that disrupting the CNBHD-CNBHD interface shifts the conformational equilibrium away from the resting state and toward activation-permissive states. Based on the rotation model of KCNH channel activation supported by recent structural studies [4,5,62], this shift may arise because disruption of the CNBHD-CNBHD interface promotes partial rotation of the intracellular gating ring, thereby favoring such pre-open states. Such a mechanism would account for the hyperpolarizing shift in activation, the accelerated activation, and deactivation kinetics observed in mutant channels. Intriguingly, several gain-of-function mutations in human EAG1 channels associated with Temple-Baraitser and related neurodevelopmental syndromes exhibit remarkably similar gating phenotypes, including hyperpolarized activation and accelerated channel opening [49]. Moreover, one of these disease-causing variants alters a conserved proline residue in the intracellular C-linker not far from the CNBHD-CNBHD interface. Although the structural consequences of these mutations remain unknown, these observations raise the possibility that disruption of interactions between adjacent CNBHDs may represent a common mechanism underlying a subset of KCNH-associated channelopathies. Future structural, kinetic, and computational studies will be required to test this hypothesis and determine how interactions between adjacent CNBHDs shape the energy landscape of KCNH channel gating.

In summary, our findings identify EGL-2 as a new regulator of sleep and reveal a conserved role for the CNBHD-CNBHD interface in restraining KCNH channel activation. Mutations at this interface enhance the function of EGL-2, UNC-103, and hERG, demonstrating that interactions between adjacent CNBHDs contribute to channel gating across multiple KCNH family members. More broadly, our work identifies the CNBHD-CNBHD interface as a previously unrecognized element of the KCNH gating machinery and provides a framework for understanding how structural rearrangements within the intracellular gating ring regulate channel opening and closing.

## Materials and methods

### *Caenorhabditis elegans* strain culture

All *C. elegans* strains used in this study were maintained at room temperature on 60 mm nematode growth media (NGM) plates seeded with *E*. *coli* OP50. A detailed list of strains used in this study is included in S1 Table.

### Molecular biology

Plasmids used in this study were constructed using standard molecular cloning techniques, including Gibson assembly, SapTrap (a type of Golden Gate cloning), and site-directed mutagenesis (New England Biolabs, Ipswich, MA). DNA sequences in plasmids were verified via Sanger sequencing (Functional Biosciences, Madison, WI) or Oxford Nanopore long-read sequencing (Plasmidsaurus, Eugene, OR).

The *egl-2(+)* cDNA was amplified from pTG44 (a gift from Rene Garcia [63],), and a silent mutation was introduced to disrupt a SapI restriction enzyme site for subsequent SapTrap cloning. All amino acid positions of EGL-2 described in this paper correspond to the *egl-2* isoform a (*GenBank accession No.* NM_071001.5). The *rgef-1* promoter [64], the *mlc-2* promoter [65], a synthetic promoter containing the *nlp-67* intron sequence plus the *pes-10* mini promoter, and the *flp-11* promoter [66] were used to drive expression of *egl-2* specifically in neurons, muscles, ALA, and RIS, respectively. Our observations showed that the *nlp-67* promoter drives expression specifically in the ALA neuron; a detailed characterization of the expression and function of *nlp-67* will be reported in a separate manuscript.

The codon-optimized GCaMP6s sequence for expression in *C. elegans* was amplified from the plasmid pHJL1 (a gift from Hang Lu, Addgene plasmid # 199376 [67],) to construct the single-copy insertion plasmid pHW1926 (*Pflp-24::GCaMP6s::SL2::mCherry::tbb-2 3’UTR*) for specific expression in the ALA neuron using the *flp-24* promoter ([23]). A detailed list of plasmids and oligos used in this study is included in S2 and S3 Tables.

### Transgenesis

The standard microinjection procedure for *C*. *elegans* was used to inject DNA into the gonads of adult hermaphrodites to generate transgenic worms [68]. Several specific transgenic strategies used in this study are described below.

Extrachromosomal arrays for *egl-2* genomic DNA rescue were constructed by injecting a solution mixture containing 20 ng/µL of the fosmid (WRM0641cC05), which contains the whole genomic region of *egl-2*, 40 ng/µL of the co-injection marker plasmid KP708 (*Pttx-3::DsRed2::unc-54 3’UTR*), and 40 ng/µL of the plasmid pBluescript KS (+) as DNA filler into the strain ZZZ944 *syIs231 II; egl-2(sy985) V*.

Single-copy transgenes were generated using Flp recombinase-mediated cassette exchange (RMCE) or rapid RMCE [69,70]. RMCE constructs and rapid RMCE constructs were built in the vectors pNM3643 and pNM4561 (gifts from Michael Nonet), respectively. Each final construct was injected at 50 ng/µL into a strain containing both a landing site and a germline-expressing Flp transgene. The ALA neuron GCaMP6s line was obtained by inserting *Pflp-24::GCaMP6s::SL2::mCherry::tbb-2 3’UTR* into the landing pad *jsSi1691 II* using RMCE. The *Pnlp-67::GFP1–10::tbb-2 3’UTR* strain and all other single-copy insertions for tissue- or cell-specific expression of *egl-2* were generated by inserting corresponding transgenes into the landing pad *jsSi1988 IV* using rapid RMCE. The strains generated in this study by rapid RMCE carried a fluorescent marker (*Pcup-4::mScarlet*) and a *hygR* gene, which is flanked by directly repeated loxP sites and can be excised by Cre recombinase [70]. For the calcium imaging experiment, the *egl-2(+)* rescue transgene (*tanSi373*) was obtained by direct injection of a Cre-expressing plasmid into the parental strain ZZZ2022 to remove the loxP-flanked marker sequence in *tanSi359*, generating the final strain ZZZ2048. The Cre injection solution mixture contained 25 ng/µL of pMDJ39 (*Peft-3::Cre::tbb-2 3’UTR*; a gift from Christian Froekjaer-Jensen; Addgene plasmid # 191381 [71],), 25 ng/µL of the co-injection marker plasmid pMH165 (*Plin-44::mCherry::tbb-2 3’UTR*; a gift from Derek Sieburth), and 50 ng/µL of the plasmid pBluescript KS (+) as DNA filler.

The endogenous tagging *egl-2::7x gfp11* strain was created using a previously described CRISPR/Cas9 knock-in method via the self-excising cassette (SEC) selection [72], with slight modification. Briefly, a solution mixture containing 5 ng/µL of the Cas9 plasmid pDD121, 5 ng/µL of the guide RNA plasmid pHW1198, 25 ng/µL of the repair template plasmid pHW2001, and 7.5 ng/µL of two red co-injection markers (2.5 ng/μL of pHW1385 *Pmyo-2::HisCl1::SL2::mScarlet-I-C1::tbb-2 3’ UTR* and 5 ng/μL of pHW1391 *Psnt-1::HisCl1::SL2::mScarlet-I-C1::tbb-2 3’ UTR,* [73]) was injected into N2 worms. Positive selection with hygromycin and negative selection with histamine were applied sequentially three and six days after injection, respectively, to select for candidate knock-in lines. Successful insertion of *7x gfp11* at the 3’ end of *egl-2* in the final strain, after removing SEC, was validated by sequencing a PCR product containing the entire insert as well as the homology arms.

Point mutations and deletions in endogenous *egl-2* and *unc-103* genomic loci were generated using protein-based CRISPR/Cas9 genome editing with the *dpy-10* co-conversion marker [31,74]. Briefly, a solution mixture of Cas9 protein (QB3 MacroLab, UC Berkeley), tracrRNA (Alt-R CRISPR-Cas9 tracrRNA, 20 nmol, IDT), gRNAs for the targeted gene and *dpy-10* (Alt-R CRISPR-Cas9 crRNA, 2 nmol, IDT), and ssDNA repair templates (Ultramer DNA Oligo, 4 nmol, IDT) was injected. F_1_ progeny with the *dpy-10* marker were singled out and genotyped to identify potential successful edits. All the desired mutations and deletions in the final strains were confirmed by sequencing. The number of residues mutated in *egl-2(G574)* and *unc-103(G427)* corresponds to *egl-2* isoform a (*GenBank accession No.* NM_071001.5) and *unc-103* isoform f (*GenBank accession No.* NM_001381823.1), respectively. A list of gRNAs, repair templates, and genotyping primers for each CRISPR/Cas9-generated strain is included in S3 Table.

### Stress-induced sleep assays

Two sleep assays were performed as previously described, with slight modifications [21]. (1) Endogenous stress-induced sleep (SIS) using heat-shock stress: strains without the *syIs231(HS::EGF)* transgene were used. One-day-old adult animals were heat shocked at 35 °C for 30 min in a Precision circulating water bath (Thermo Scientific, CIR19). The entire 60 mm NGM plate containing the worms was immediately placed into ice for 2 min to cool down and then returned to room temperature. Sleep behavior was scored every 15 min following heat shock. (2) EGF-induced sleep assay: strains carrying the *syIs231(HS::EGF)* transgene were used. One-day-old adult hermaphrodites were heat shocked at 33 °C for 15 min in an Isotemp circulating water bath (Fisher Scientific, 4100 H19A) to transiently induce EGF overexpression from the *syIs231* transgene. The worms were immediately returned to room temperature. Sleep behavior was scored 2 h post-heat shock. At this time point, the worm would have recovered from endogenous behavioral quiescence due to the mild heat stress and would only show sleep resulting from the transgene overexpression [22]. Sleep behavior was scored based on behavioral quiescence, defined as the absence of both pharynx pumping (feeding) and locomotion during a 4-s observation under a dissecting microscope [21]. An animal showing feeding or visible movement of either head or body was scored as non-quiescent. The observer was blind to the genotypes of the worms assayed while scoring.

Typically, pre-seeded 60 mm NGM plates, containing 10 mL of NGM agar, were taken out from our cold room two days before the heat-shock assay. One day later, L4 animals for each genotype were hand-picked for synchronization and transferred to these NGM plates, with 15–25 animals per plate as a biological replicate. The next day, one-day-old adults within the OP50 food lawn on each plate were examined for feeding and locomotion both before (pre) and after (post) heat shock. After pre-heat shock scoring, each plate was parafilmed and placed to float on the surface of the circulating water heat bath at the temperature and duration specified above. During heat shock, the plates were right-side up, with the agar-containing bottom submerged in the water. Upon returning to room temperature, the parafilm was removed, the lid was wiped dry and left off for 5 min, and the plates were left unperturbed with the lid on until scoring sleep phenotypes. Each sleep assay was performed in three biological replicates.

### Forward genetic screen and genetic mapping

*egl-2(sy985)* was isolated in a suppressor screen for mutants defective in EGF-induced sleep. Ethyl methanesulfonate (EMS) mutagenesis, the forward genetic screen, and the mapping step were performed as previously described [75], with slight modifications. Briefly, L4 worms from the parental strain PS7244 *syIs231(HS::EGF)* were treated with 50 mM EMS (Sigma-Aldrich, St. Louis, MO) for 4 h to induce random mutations. After recovery, several P_0_ worms were placed on NGM plates. 3–4 days later, gravid adult F_1_ worms from each plate were lysed to release eggs, which were placed on new plates. The resultant semi-synchronized F_2_ worms were screened to identify sleep-defective mutants that did not exhibit behavioral quiescence between 2 and 3 h after 15-min heat shock. *egl-2(sy985)* was mapped and cloned using a one-step whole-genome sequencing and SNP mapping strategy [76]. Briefly, the *syIs231; egl-2(sy985)* mutant was crossed with the Hawaiian strain CB4856. Individual F_2_ progeny defective in EGF-induced sleep were selected and singled out. Worms from 34 F_2_ plates, in which the mutant phenotype was confirmed in the F_3_ generation, were pooled to extract genomic DNA, which was used for whole-genome sequencing with paired-end 150-bp reads at about 30x coverage (Azenta Life Sciences, South Plainfield, NJ). The sequencing result was analyzed and compared to the *C. elegans* reference genome (ce10) using MiModD v0.1.9 [77]. A G-to-A transition at chrV:1,292,033 was identified in the coding region of *egl-2 (F16B3.1)*, which is predicted to cause a nonsynonymous Gly574Glu (G574E) substitution. This variant was confirmed by Sanger sequencing a PCR product amplified from *egl-2(sy985)* genomic DNA. The sequence of the *egl-2(sy985)* allele is CAATGAGCACAGTTGTTTTCGTCTTGCATCTGACGaGTGTCTGCGGTCTCTTGCGATGTTTCTGGAGCTGA, including 35 bp of wild-type flanking sequence on each side, with the mutated nucleotide in lowercase.

### Egg-laying assay

Egg-laying behavior was assessed using a previously described protocol that measures the number of eggs retained in the uterus [37]. Briefly, animals were synchronized by handpicking late L4 worms and assayed 30 h later. Individual adult worms were lysed to release unlaid eggs from the uterus by transferring each worm into a 7-µL drop of 20% commercial bleach (Clorox) on the lid of a 96-well plate. 10 min after bleaching, the number of eggs in each drop was quantified by direct observation under a standard dissecting microscope at 50x magnification. A total of 30–50 animals were analyzed per strain across three biological replicates.

### Microscopy

Worms were transferred to a 15-µL drop of 30 mg/mL 2,3-butanedione monoxime (BDM) solution (Sigma-Aldrich, St. Louis, MO) on a coverslip and washed with the same BDM solution several times to remove OP50 bacteria, until fully immobilized. Animals were then mounted by putting a freshly prepared 2% agarose pad on a glass slide onto the coverslip with a drop of BDM solution containing the immobilized worms. All images were captured using a Nikon Ti2-E Eclipse inverted microscope connected to a Prime 95B (25mm) sCMOS camera and a SPECTRA X LED light engine (Lumencor, Beaverton, OR). The entire imaging system was controlled by the NIS-Elements Advanced Research software (Nikon).

Imaging EGL-2 expression in the ALA neuron was performed in worms at the L4 stage, during which the cell body of the ALA neuron can be reliably identified using DIC (differential interference contrast) microscopy. DIC and GFP fluorescence micrographs were captured using a Nikon Plan Apochromat VC 100X/1.40 oil objective. All images were oriented with the anterior to the left and the ventral side down. To quantitatively compare EGL-2 expression levels in wild-type and *egl-2* mutants, a region of interest (ROI) was manually drawn around the cell body of the ALA neuron (NIS-Elements Advanced Research software, Nikon). A second ROI of the same size was duplicated and placed in an adjacent background region. GFP fluorescence intensity within the ALA neuron was calculated by subtracting the mean background intensity from the mean intensity of the ALA neuron. A total of 30–40 worms were analyzed per strain in three biological replicates.

Imaging eggs within the uterus of *unc-103* mutants was performed in synchronized adult animals 30 h after the late L4 stage. Brightfield micrographs were captured using a Nikon Plan Apochromat Lambda 10X/0.45 dry objective.

### *In vivo* calcium imaging

Since the ALA neuron is also a mechanosensor that can be activated by strong mechanical stimuli [78], Ca^2+^ imaging of ALA neuronal activity was performed in freely moving worms. *lite-1 gur-3* mutations were included in all strains to reduce blue light-induced movement of the worms during imaging [79,80]. Worms were imaged without heat shock or 15 min after a 30-min heat shock in a 35 °C water bath, following the SIS heat shock protocol described above. After heat shock, the NGM plates were put on ice for 2 min to cool down, and the lid was off for 8 min till the worm samples were loaded for imaging, as described below.

One day before imaging, 60 mm NGM plates were seeded with 50 uL *E. coli* OP50 culture at the center. The plates were stored at room temperature overnight to grow a thin layer of OP50. Meanwhile, L4 worms were hand-picked to synchronize. The next day, 2–3 one-day-old adults were transferred to each NGM plate and allowed to rest for at least 1 h to let the ALA neuron recover from any potential activation due to the mechanical stimulus introduced during transfer using a platinum wire. To minimize potential mechanical stimuli from the coverslip during imaging, worms were sandwiched between the coverslip and the NGM agar, with a 100-nm-thick PDMS film in between as a spacer. Specifically, a PDMS film with a circular hole of 8 mm in diameter was gently placed onto the NGM plate to trap 1–3 worms; a chunk of NGM was then transferred to a coverslip (60 mm x 24 mm), with the side containing the PDMS film and worms facing the coverslip. A 22 mm x 22 mm coverslip was placed on the other side of the NGM chunk to reduce evaporation. Dual-channel recordings of the samples were captured using the Nikon Ti2-E Eclipse inverted microscope equipped with an image splitter (OptoSplit II from Cairn Research), a Prime 95B (25mm) sCMOS camera and a Nikon 10X/0.45 dry objective. GCaMP6s and mCherry were excited at 470 nm (10% blue light) and 575 nm (20% yellow light), respectively. Videos were captured with a 25-ms exposure time for 30 frames at 2x2 binning.

To analyze the fluorescence intensity, the cell body of the ALA neuron was detected as the ROI in each video using spot detection (NIS-Elements Advanced Research software, Nikon). For each frame, the fluorescence intensity of the ALA neuron in each channel was quantified by subtracting the mean background intensity from the mean intensity within the ROI. The GCaMP6s/mCherry ratio was then calculated for that frame. The average GCaMP6s/mCherry ratio across all 30 frames was used as the value for that recording.

### *Xenopus* oocyte expression and electrophysiology

*Human ERG1a* (*hERG1* or *KCNH2*; *GenBank accession No.* NM_000238.4) cDNA was cloned into the pGH19 oocyte expression vector with the T7 promoter. The G749R mutation in *hERG1a* cDNA was generated using site-directed mutagenesis (New England Biolabs) and verified by DNA sequencing (Plasmidsaurus). Each plasmid DNA was linearized with the restriction enzyme NotI, purified, and then transcribed using the mMESSAGE mMACHINE T7 Transcription Kit (Invitrogen, AM1344). The cRNA was purified using the MEGAclear Transcription Clean-Up Kit (Invitrogen, AM1908).

*Xenopus laevis* oocytes were first isolated and treated with 75 µg/ml Liberase (Thermolysin Medium) research-grade enzyme (Sigma-Aldrich) for 30 min in Ca^2+^-free ND96 solution (96 mM NaCl, 2 mM KCl, 1 mM MgCl_2_, and 5 mM HEPES, with pH adjusted to 7.4 with NaOH) to remove the follicular membranes. Defolliculated oocytes were injected with 1.5 ng wild-type or G749R cRNA and incubated for 2 d at 19 °C in storage solution (96 mM NaCl, 2 mM KCl, 1.8 mM CaCl_2_, 1 mM MgCl_2_, 1 mM HEPES, and 10 mg/L gentamicin, with pH adjusted to 7.4 with NaOH) before voltage-clamp recording.

Two-electrode voltage-clamp recordings were performed on the oocytes in recording solution (96 mM NaCl, 2 mM KCl, 0.3 mM CaCl_2_, 1 mM MgCl_2_, and 5 mM HEPES, with pH adjusted to 7.4 with NaOH). Glass microelectrodes were filled with 2 M KCl and had resistances of 3–10 MΩ. Digidata 1440A data acquisition system and pCLAMP software (Molecular Devices) were used in recording with command protocols. To determine the I-V relationship, oocytes were initially held at -80 mV and then applied with a series of depolarization potentials ranging from -100 mV to +40 mV in 10 mV increments. Each step lasted 1000 ms and was followed by a repolarization step at -105 mV for 2000 ms. Conductance and voltage data were fitted to a single Boltzmann function: $\begin{array}{c} I\;/\;I_{max}=\left(I_{min}\right)+\frac{{(I}_{max}\;-\;I_{min})}{\text{1}\;+\;\text{exp}\left(\frac{V_{\text{1}/\text{2}}\;-\;V}{k}\right)} \end{array}$, where$\;V_{\text{1}/\text{2}}$ is the half-activation potential, V is the test voltage, and k is the slope factor.

Activation kinetics were assessed using an envelope-of-tails protocol [38]. From a holding potential of −80 mV, channels were depolarized to +20 mV for progressively increasing durations in 25 ms increments, then repolarized to −105 mV to elicit tail currents. Peak tail current amplitudes were extracted from individual traces, plotted as a function of depolarization duration, and fitted with a single-exponential function to determine the activation time constant (𝛕). For Fig 8G, peak tail currents were normalized to the maximal current for each oocyte, averaged within each group, and fitted with a single-exponential function.

For measuring deactivation kinetics, tail currents were recorded at −105 mV after channel activation at +40 mV. Deactivation time constants (𝛕) were determined by fitting the decaying phase of the tail currents with a single-exponential function.

Two independent electrophysiological experiments were performed on different days, with 9–10 oocytes recorded per condition in each experiment. Both experiments yielded similar results. The data shown in Fig 8 are from one representative experiment.

### Experimental design and statistical analysis

Behavioral experiments were conducted in three replicates. Age-synchronized hermaphrodite *C. elegans* were used to reduce variability. Raw data for all behavioral assays can be found in S1 Data. Statistical tests were performed using GraphPad Prism (Dotmatics) with unpaired t-tests for comparing two groups or one-way ANOVA for comparing more than two groups. Details of statistical tests used for individual results are listed in the figure legends.

### Use of artificial intelligence tools and technologies in the writing process

During the preparation of this manuscript, the authors used Copilot and ChatGPT to improve the fluency and clarity of the writing. After using these tools, the authors reviewed and edited the content as needed and take full responsibility for the content of the published article.

## Supporting information

S1 Fig Conservation of the core architecture of EAG potassium channels. Alignment of the protein sequences spanning the region from the PAS domain to the cyclic nucleotide-binding homology domain (CNBHD) of EAG channels from the following species: *Caenorhabditis elegans* (EGL-2, NP_503402.3)*, Mus musculus* (mEag1, NP_034730.1), *Rattus norvegicus* (rEag1, NP_113930.1), *Homo sapiens* (hEAG1, NP_758872.1), *Xenopus laevis* (fEAG1, XP_041418453.1), and *Drosophila melanogaster* (dEAG, NP_001036275.1). Conserved residues are highlighted in black. Functional domains are indicated above the alignment, including the PAS domain, the voltage sensor domain (S1–S4), the pore-forming segments (S5–S6), the potassium ion selectivity filter (SF), the CNBHD, and the intrinsic ligand motif. Residue numbering at the top corresponds to EGL-2 isoform a. The red star indicates the highly conserved glycine residue that is mutated in *egl-2(sy985)* and *egl-2(tan200)*. C-terminal sequences to the intrinsic ligand are omitted.
(TIF)

S2 Fig Conserved CNBHD-CNBHD interface in EAG and ERG channels. Sphere representations of the intracellular gating ring of the following three homotetrameric channels: *C. elegans* EGL-2 (AlphaFold3-predicted structure), rEag1 (cryo-EM structure; PDB: 5K7L), and hERG (cryo-EM structure; PDB: 5VA1). The PAS domains and CNBHDs from each of the four subunits are colored yellow, green, orange, and purple; other domains are omitted for clarity. The EGL-2 G574 residue and the corresponding residues rEag1 G561 and hERG G749, highlighted in red, are all located at the conserved CNBHD-CNBHD interface. (**A, C, E**) Bottom views of the intersubunit structural arrangement of the PAS domain and CNBHD within the intracellular gating ring of (A) EGL-2, (C) rEag1, and (E) hERG. The CNBHDs form an inner ring, with PAS domains positioned peripherally. (**B, D, F**) Side views of two CNBHDs from adjacent subunits of (B) EGL-2, (D) rEag1, and (F) hERG, generated by a 90° downward rotation of the bottom views shown in (A, C, E) and displayed at higher magnification, with the PAS domains omitted for clarity. Top, opaque sphere representation of two adjacent CNBHDs; bottom, a magnified view of a smaller region containing the CNBHD-CNBHD interface at 70% transparency, for better visualization of the conserved glycine residue (in red) at the interface.
(TIF)

S3 Fig *egl-2(G574E)* and *egl-2(G574R*) mutants are defective in egg laying. Quantification of the eggs retained in the uterus of adult animals with the indicated genotypes. Numbers shown on the bars indicate the total number of animals scored for each genotype. The results are consistent with the idea that *egl-2(G574R)* is a stronger gain-of-function allele than *egl-2 (G574E)*. Mean ± SEM are shown. ****, p < 0.0001. One-way ANOVA with Dunnett’s multiple-comparisons test.
(TIF)

S1 Video Location of G574 at the CNBHD-CNBHD interface of the EGL-2/EAG channel. A rotating view of structural models of the PAS domains and CNBHDs within the EGL-2/EAG tetramer channel is followed by a magnified view of the residue G574 at the CNBHD-CNBHD interface. PAS domains are shown in orange, CNBHDs in cyan, and G574 in red. The structure was predicted using AlphaFold3. Other domains of EGL-2 are omitted for clarity.
(MP4)

S1 Table Strains used in this study. (XLSX)

S2 Table Plasmids used in this study. (XLSX)

S3 Table Oligos used in this study. (XLSX)

S1 Data Raw data for all behavioral assays. (XLSX)
