# Supplementary material for: Forward genetics reveals a conserved CNBHD-CNBHD interface that restrains EAG channel activation to regulate sleep
Source: PLoS Genet. 2026 Oct 1;22(10):e1012255. doi: 10.1371/journal.pgen.1012255 (PMC13660981; doi:10.1371/journal.pgen.1012255)
Supplement: Supplementary file 1 [file pgen.1012255.s010.pdf]

Response to reviewers' comments (PGENETICS-D-26-00728)

We sincerely thank the editor and three reviewers for their constructive and insightful comments. We have carefully considered all comments and revised the manuscript accordingly. The major revisions include: 1) adding descriptions of some additional phenotypes observed in *egl-2(G574E) and egl-2(G574R)* mutants, 2) providing better illustrations of the CNBHD-CNBHD interface, and 3) revising the Abstract, Discussion, and Methods to address reviewers' comments. We believe that these changes have strengthened the manuscript.

Kindly note that our point-by-point responses are provided below (in blue).

Response to Reviewer #1:

Reviewer #1: Starting from a forward genetics screen for sleep mutants in C. elegans Huang and colleagues describe the effect of a conserved glycine mutation (G574E) In the EGL-2 potassium channel on sleep in C. elegans. They use a combination of forward discovery genetics, in vivo and in oocytes structure-function analysis, cell specific rescue experiments, and calcium imaging in live behaving animals. They make use of Xenopus oocyte electrophysiology to perform 2 electrode voltage clamp experiments in order to characterize the biophysical changes conferred by the channel mutation. They conclude that the G574E mutation results in a "gain of function" effect both in vivo and in cellulo. The effect of the mutated channel maps to the ALA neuron.

This paper would be of interest to sleep researchers, ion channel physiologists, behavioral geneticists, and human geneticist who study potassium channelopathies.

The experiments are technically sound, the writing is clear, and the data are presented logically. I have only minor comments.

We are pleased that this reviewer found our paper of interest, and we sincerely thank the reviewer for the positive evaluation and thoughtful comments.

1. The authors make a strong case for G574E being a gain-of-function allele of egl-2. However, in figure 1, they show that introducing a wild-type copy of egl-2 into the egl-3(G574E) mutant fully rescues the sleep defect. This does not bother me (as I can imagine that the excess wild-type copies in the transgene array could compete away the channels with elevated potassium conductance), but should be commented on

We appreciate this reviewer for this insightful comment. We agree with the reviewer's interpretation for two main reasons: 1) The rescue transgene arrays in Figure 1 contain many copies of the *egl-2(+)* fosmid, which are expected to overexpress wild-type EGL-2 protein; 2) EGL-2 channel functions as a tetramer. To address this point while maintaining the flow of the paper, we have included additional text in the Discussion section of the revised manuscript, instead of the Results section, since in Figure 1, it is still not yet clear that *egl-2(G574E)* is a gain-of-function allele.

Revised text (Lines 383–388).
"Interestingly, although *egl-2(G574E)* is a gain-of-function allele, expression of wild-type *egl-2* rescued the sleep defects in *egl-2(G574E)* mutants (Fig 1C). Given that EGL-2 channel is a tetramer, overexpression of the wild-type EGL-2 subunit from the rescue transgenes likely

dilutes the mutant EGL-2(G574E) subunit during channel assembly, dramatically reducing the fraction of fully assembled EGL-2(G574E) channels and thus rescuing the sleep defects."

2. They provide evidence that G574E is acting specifically in the ALA sleep-promoting neuron to reduce its excitability. But since egl-2 is expressed widely, I am surprised the mutant does not show more pleiotropic phenotypes such as locomotion, egg laying, pharyngeal pumping, and defecation defects. The authors should comment on this apparent lack of pleiotropy.

We apologize for not describing additional phenotypes in the original manuscript, which may have led to the misunderstanding that the G574E mutation only acts in the ALA neuron to impact sleep, without other pleiotropic phenotypes. Indeed, *egl-2(G574E)* mutants had other behavioral phenotypes, including defects in locomotion and egg-laying. Accordingly, we have included additional text in the Results section of the revised manuscript and included one Supplementary figure (Figure S3) to show some additional behavioral phenotypes observed in *egl-2(G574E)* and *egl-2(G574R)* mutants.

Revised text (Lines 258–265).
"In addition, similar to previously described gain-of-function *egl-2* mutants [26], both *egl-2(G574R)* and *egl-2(G574E)* mutants exhibited other behavioral phenotypes, including abnormal locomotion and defective egg laying. Notably, heterozygous *egl-2(G574R)* mutants showed more severe sleep defects than heterozygous *egl-2(G574E)* mutants (Fig 6B), while homozygous *egl-2(G574R)* retained more eggs than *egl-2(G574E)* mutants (S3 Fig). Together, these results indicate that *egl-2(G574R)* is a stronger gain-of-function allele than *egl-2(G574E)*."

3. Were the G574E mutants defective in sleep induced by other stressors? E.g. UVC and Cry5B.

We had very preliminary data showing G574E and G574R were partially defective in SIS induced by UV and Cry5B, consistent with the idea that gain-of-function *egl-2* suppresses sleep by inhibiting the activity of the ALA neuron. In the current study, we focus on using heat shock as the main stressor paradigm. However, we agree that in follow-up studies, it can be valuable to rigorously test whether *egl-2(gof)* mutants are also SIS included by other cellular stressors, including UV and Cry5B mentioned.

4. The authors propose that their results show a role for potassium channels in sleep. But, they only saw phenotypes with the gof mutant. The lof had no apparent phenotype in their experiments. Hence, it is unclear to what degree EGL-2 wt protein (i.e. one that is not mutated) is involved in normal sleep regulation.

We thank this reviewer for this insightful comment. The *C. elegans* genome has about 70 potassium channel genes, many of which likely function redundantly to control neuron excitability. Loss-of-function mutations in many individual potassium channel genes do not lead to obvious phenotypes, while gain-of-function mutations usually cause dramatic behavioral phenotypes (PMCID: PMC4781360). As previously reported (PMCID: PMC6782970), *egl-2(lof)* mutants do not have gross behavioral defects (e.g., locomotion and egg laying). Thus, it is not entirely surprising that *egl-2(lof)* had no apparent sleep phenotype. Our results are consistent with the idea that EGL-2 probably functions with other potassium channels to control the excitability of the ALA neuron for sleep regulation. Accordingly, we have included additional text in the Discussion section of the revised manuscript to address this point.

Revised text (Lines 388–397).

“It is also worth noting that no obvious abnormal sleep phenotypes were observed in *egl-2* loss-of-function alleles in our assays (Figs 4B-4D), consistent with previous observations that animals lacking *egl-2* have grossly normal behavioral phenotypes [26]. We speculate that, in wild type, EGL-2 functions redundantly with other potassium channels to control proper excitability of the ALA neuron. Indeed, the *C. elegans* genome encodes about 70 potassium channel genes, many of which are expressed in the ALA neuron, as detected by single-cell RNA-seq [23,44,45]. The lack of abnormal sleep phenotypes in *egl-2* loss-of-function alleles may reflect functional compensation by other potassium channels in the ALA neuron in the absence of *egl-2*.”

5. The authors use the nlp-67 promoter in order to express transgenes specifically in the ALA neuron. Can they provide data or a reference that shows this specificity?

We thank this reviewer for pointing this out. The ALA-specific expression of the *nlp-67* promoter used in this study is based on our unpublished data from a separate project focused on the expression and function of *nlp-67*, for which we are currently preparing a manuscript for publication. The specific promoter we used drives expression in a single neuron in the head region that matches the location of the cell body of ALA neuron, which was further confirmed by a co-colorization experiment. We will add the reference if the *nlp-67* manuscript is available on BioRxiv before the publication of the *egl-2* paper. Meanwhile, we have included additional text in the Methods of the revised manuscript to clarify this point and hope that this addresses this comment.

Revised text (Lines 498–503).
“The *rgef-1* promoter [62], the *mlc-2* promoter [63], a synthetic promoter containing the *nlp-67* intron sequence plus the *pes-10* mini promoter, and the *flp-11* promoter [64] were used to drive expression of *egl-2* specifically in neurons, muscles, ALA, and RIS, respectively. Our observations showed that the *nlp-67* promoter drives expression specifically in the ALA neuron; a detailed characterization of the expression and function of *nlp-67* will be reported in a separate manuscript.”

6. I would like to see more details on their method for heat shocking as this assay is notoriously noisy/variable. Specifically, did the worms have access to ample food during the heat shock? Were the plates immersed in the water bath right-side up or upside down? What was the volume of NGM agar on the plate? How old were the NGM plates?

We apologize for not describing the heat shock protocol well in the original manuscript. We also recognized that the SIS assay using the heat shock protocol can sometimes be noisy. The *egl-2 (gof)* phenotype was robust, and we have been performing independent repeats for a particular experiment within a couple of days using similar plates with ample food. As suggested, we have included additional details of the heat shock protocol in the Methods section of the revised manuscript.

Revised text (Lines 567–597).
“Two sleep assays were performed as previously described, with slight modifications [21]. (1) Endogenous stress-induced sleep (SIS) using heat-shock stress: strains without the *syIs231(HS::EGF)* transgene were used. One-day-old adult animals were heat shocked at 35 °C for 30 min in a Precision circulating water bath (Thermo Scientific, CIR19). The entire 60 mm NGM plate containing the worms was immediately placed into ice for 2 min to cool down and then returned to room temperature. Sleep behavior was scored every 15 min following heat shock. (2) EGF-induced sleep assay: strains carrying the *syIs231(HS::EGF)* transgene were

used. One-day-old adult hermaphrodites were heat shocked at 33 °C for 15 min in an Isotemp circulating water bath (Fisher Scientific, 4100 H19A) to transiently induce EGF overexpression from the *syIs231* transgene. The worms were immediately returned to room temperature. Sleep behavior was scored 2 h post-heat shock. At this time point, the worm would have recovered from endogenous behavioral quiescence due to the mild heat stress and would only show sleep resulting from the transgene overexpression [22]. Sleep behavior was scored based on behavioral quiescence, defined as the absence of both pharynx pumping (feeding) and locomotion during a 4-s observation under a dissecting microscope [21]. An animal showing feeding or visible movement of either head or body was scored as non-quiescent. The observer was blind to the genotypes of the worms assayed while scoring.

Typically, pre-seeded 60 mm NGM plates, containing 10 mL of NGM agar, were taken out from our cold room two days before the heat-shock assay. One day later, L4 animals for each genotype were hand-picked for synchronization and transferred to these NGM plates, with 15-25 animals per plate as a biological replicate. The next day, one-day-old adults within the OP50 food lawn on each plate were examined for feeding and locomotion both before (pre) and after (post) heat shock. After pre-heat shock scoring, each plate was parafilmed and placed to float on the surface of a circulating water heat bath at the temperature and duration specified above. During heat shock, the plates were right-side up, with the agar-containing bottom submerged in the water. Upon returning to room temperature, the parafilm was removed, the lid was wiped dry and left off for 5 min, and the plates were left unperturbed with the lid on until scoring sleep phenotypes. Each sleep assay was performed in three biological replicates."

7. The authors use the term "standard dissecting microscope". Is the word "standard" necessary?

As suggested, we have removed the word "standard".

8. Why was imaging egl-2 in the ALA neuron performed in the L4 stage? Why not in adults?

The fluorescence intensity of EGL-2 fusion in the ALA neuron is dim. For practical reasons, it is much easier and more reliable to identify the location of the ALA neuron at the L4 stage for the quantitative imaging experiment to compare EGL-2 expression across different genotypes using high magnification fluorescent microscopy (Figure 2B and Figure 6D, 100x objective). We have added additional text in the Methods section to clarify this point.

Revised text (Lines 647–649).
"Imaging EGL-2 expression in the ALA neuron was performed in worms at the L4 stage, during which the cell body of the ALA neuron can be reliably identified using DIC (differential interference contrast) microscopy."

Response to Reviewer #2:

Reviewer #2: In the manuscript, "Forward genetics reveals a conserved CNBHD-CNBHD interface that restrains EAG channel activation to regulate sleep", the authors identified a function for the cyclic nucleotide binding homology domain in the C. elegans-encoded EGL-2 voltage-gated K+ channel. The authors used the mutagen EMS to induce point mutations into the C. elegans genome, and then they conducted a nice forward genetics screen to identify a mutation that interfered with the ALA mechanosensory interneuron's function to promote behavioral quiescence after heat-shock induction of LIN-3/EGF.
The authors found that an EMS-induced mutation changed a conserved glycine to a glutamate

amino acid in the cyclic nucleotide binding domain of EGL-2, causing the channel to show phenotypic properties reminiscent of unregulated K+ conduction. The experiments presented in the results sections convincingly demonstrate that the mutant gain-of-function (gf) allele promotes premature and/or excessive EGL-2 -mediated K+ conduction, which attenuates depolarization of the ALA, and consequently interferes with heat-shock LIN-3 expression- or experimental heat stressed-induced behavioral quiescence.
The authors further explored the function of EGL-2's CNBHD region by introducing additional missense mutations in the CNBHD and the intrinsic ligand domain. From their well-controlled experiments, they conclude that interactions between CNBHD and the intrinsic ligand domain regulate the activation and deactivation kinetics of ion conduction.
Finally, the authors demonstrated that their findings apply to other voltage-gated KCNH channels. They introduced the CNBHD gain-of-function mutation into the C. elegans ERG homolog UNC-103 and in the human K+ channel hERG1a. From an egg-laying behavioral assay, the authors showed that mutant UNC-103, containing the CNBHD (gf) allele, reduced the frequency of egg-laying muscle contractions. Additionally, from electrophysiological studies using xenopus oocytes, the authors showed that mutant hERG, containing the CNBHD (gf) allele, display accelerated K+ conductance at more negative voltages.
The experiments, detailed in the manuscript, were well-executed and conservatively interpreted. The identification of the gain-of-function allele underscores the utility of using chemical-mediated random mutagenesis and forward genetic screens to uncover functions for distinct domains in biomolecules; the conclusions from the experiments in this manuscript provide an important understanding on how a specific region at the interface of the intracellular cyclic nucleotide binding domains between KCNH subunits regulate the gating of ion conductance through the S5 and S6 transmembrane helixes. The manuscript was an easy read for me, and I do not have any significant issues with the research presented in the paper.
Signed, L. Rene Garcia

We sincerely thank Dr. Garcia for his enthusiasm for our manuscript and for recognizing the impact and quality of our study.

Minor comments:
1. In the abstract, page 2 line 33-34. The sentence, "In vivo calcium imaging showed that the gain-of-function mutation inhibits ALA neuronal activity to suppress sleep, consistent…", can be misread by the casual reader as if the ALA normal function is to suppress sleep, and the (gf) mutation inhibits that function. A clearer way to state this might be something like, "In vivo calcium imaging showed that the gain-of-function mutation suppresses sleep by inhibiting ALA neuronal activity, consistent with enhanced potassium channel function.

We thank the reviewer for this helpful suggestion. We agree and have used the suggested sentence in the revised manuscript (Lines 33–34).

2. In the Discussion, under the section: EAG channels and sleep regulation, one should comment in one or two sentences why in Figure 4, panel B through D, there was no obvious abnormal phenotype for reducing or eliminating EGL-2 activity on ALA's regulation of behavioral quiescence; one can mention other potential compensating K+ channels in ALA that can substitute for EGL-2 normal function.

We thank the reviewer for this insightful comment. Accordingly, we have included additional text in the Discussion section of the revised manuscript to comment on the lack of sleep abnormalities in *egl-2* loss-of-function mutants.

Revised text (Lines 388–397).
"It is also worth noting that no obvious abnormal sleep phenotypes were observed in *egl-2* loss-of-function alleles in our assays (Figs 4B-4D), consistent with previous observations that animals lacking *egl-2* have grossly normal behavioral phenotypes [26]. We speculate that, in wild type, EGL-2 functions redundantly with other potassium channels to control proper excitability of the ALA neuron. Indeed, the *C. elegans* genome encodes about 70 potassium channel genes, many of which are expressed in the ALA neuron, as detected by single-cell RNA-seq [23,44,45]. The lack of abnormal sleep phenotypes in *egl-2* loss-of-function alleles may reflect functional compensation by other potassium channels in the ALA neuron in the absence of *egl-2*."

Response to Reviewer #3:

Reviewer #3: This manuscript reports a forward genetic screen in C. elegans to identify genes involved in sleep and identified a gain of function (GOF) mutation G574E in a KCNH potassium channel, EAG (EGL-2 in C. Elegans) which is responsible for a loss of sleep, which is an interesting and novel result. G574E is located in the C-terminal CNBH Domain, is a conserved residue, and is nearby the same residue in an adjacent CNBH Domain from an adjacent subunit.

Pros

Elegantly done studies to reintroduce WT EGL-2 to mutant G574E worms and perform a Crispr introduction of G574E to WT worms to confirm the behavioral phenotype and link to G574E.

Additionally, re-introduction of EGL-2 / EAG channels with engineered point mutations that block the K ion flux out of the cell (or into the cell) indicated the EAG mutant channel effect was due to the movement of K ions.

Convincing data to show activity of the ALA sleep promoting neurons was impaired by EGL-2 G574E, consistent with GOF of a K channel, presumably due to increased membrane hyperpolarization.

To expand the scope of this finding, they also generate C. elegans in with the homologous EAG mutant in the unc103 worm (G427R), which is the locus of ERG (G749R), a channel in the same KCNH family as EAG. The unc103/ERG mutant worms have a gain of function phenotype (egg retention) in their egg laying behavior which generalizes the importance of the G574E mutant to other channels and behaviors.

The proposed function of EAG mutants as inferred by behavioral and cellular experiments is examined directly in ERG channels with the equivalent hERG G749R mutant in heterologous expression experiments. hERG G749R is shown here to have altered gating, including gain of function in some functional properties (increase in the current density and left shift of the gV). Faster deactivation in these channels, however, could be considered a LOF. All the gating effects are consistent with a disruption of the CNBHD and allosteric effects on the gating machinery.

Gain of function in channel behavior measured directly in ERG G749R is consistent with the egg laying retention and, by extension, consistent with a GOF in EGL-2 G574E, b/c a GOF would hyperpolarize and decrease ALA neuronal activity and decrease activity in the sleep promoting

ALA neurons and loss of sleep.

We are pleased that this reviewer found our study interesting and novel. We sincerely appreciate this reviewer for the positive and constructive evaluation of our manuscript.

Clarifications

Please clarify on the rationale and justification for the experiment with G574E and intrinsic ligand YNL to ANA mutants, perhaps in the Results or Discussion. Was the idea that the ANA mutant would right-shift the voltage dependence and offset the presumed left-shifted voltage dependence of G574E? The conclusion that the intact YNL is necessary for the sleep phenotype might only be true in the context of G574E (and not for instance in G574R if it has a greater GOF than G574E).

We thank the reviewer for bringing this to our attention. We apologize for not clearly explaining the rationale for the experiment with EGL-2(YNL->ANA + G547E). To address this comment, we have revised the corresponding paragraph to better explain the rationale.

Revised text in the Results section (Lines 236–253).
"Because the G574 residue lies within CNBHD, we determined whether the gain-of-function effect of EGL-2(G574E) requires a functional CNBHD. The cyclic nucleotide-binding pocket of CNBHD in KCNH channels does not bind to cyclic nucleotides but instead is occupied by an intrinsic ligand located near the end of CNBHD (Fig 5B) [7,9]. The intrinsic ligand is important for normal CNBHD function [6]. In both EAG and hERG channels, intrinsic ligand mutations cause the same channel gating defects as deletion of the entire CNBHD [12,13]. For example, in human EAG1, mutating the intrinsic ligand (YNL to ANA) and deleting the CNBHD both produce a marked right shift in the conductance-voltage relationship and slow channel activation [12]. We therefore asked whether the self-liganded CNBHD is essential for the gain-of-function phenotype observed in *egl-2(G574E)* mutants. We used CRISPR-based genome editing to engineer the endogenous *egl-2(G574E)* locus by introducing the corresponding mutations in the intrinsic ligand (residues 686-688: YNL to ANA) (Figs 4A and 5B). We found that the YNL to ANA mutations completely blocked the sleep defects observed in *egl-2(G574E)* mutants (Fig 5D). These results indicate that the gain-of-function effect of EGL-2(G574E) depends on its intact intrinsic ligand, suggesting that EGL-2(G574E) requires a functional CNBHD."

Other than the nearness at G574 in the structures and models, there is not much buried surface between CNBHDs and not much evidence that the CNBHDs of these channels interact directly. Perhaps include distance markers in the figures between the G574 and G574 to show more clearly. A change in gating with a mutant does not necessarily mean that a CNBHD-CNBHD interaction is disrupted. This result could also be consistent with a change in individual CNBHD dynamics or individual CNBHD communication with the pore. I would suggest the authors soften that conclusion a bit throughout the article.

We thank the reviewer for this insightful comment. We agree that the cartoon representation of the structure models in the original manuscript (Figure 2, Figure S2, and Video S1) do not clearly show the buried interface between two adjacent CNBHDs. Based on the Cryo-EM structures of rEag1 and hERG and the AlphaFold3-predicted structure of EGL-2, there are multiple contacts at various residues of the two CNBHDs from adjacent subunits. To better illustrate the CNBHD-CNBHD interface, we have included additional panels with sphere models of two adjacent CNBHDs of EGL-2, rEag1, and hERG (specifically, two new panels Figure 2G

and 2H (shown below) and the revised Figure S2), to highlight the conserved interface and the location of G574 within the interface.

We also agree with the reviewer that it is possible that G574E may alter individual CNBHD dynamics or individual CNBHD communication with the pore. But our data favor the interpretation that the mutations cause gain-of-function effects by disrupting the CNBHD-CNBHD interface. First, the glycine sits at the CNBHD-CNBHD interface, as shown in available Cryo-EM structures of rEag1 and hERG and the predicted structure of EGL-2. Second, both EGL-2(G574E) and EGL-2(G574R) produced similar gain-of-function phenotypes. To address this comment, we have included additional text in the Discussion section of the revised manuscript.

Revised text in the Results section (Lines 418–424).
“Without structures of the mutated EGL-2 channels, our study cannot completely rule out the possibility that the EGL-2(G574E) and EGL-2(G574R) mutations cause gain-of-function effects by altering individual CNBHD dynamics or CNBHD-to-pore communication. However, we favor the interpretation that both mutations cause gain-of-function effects by disrupting the CNBHD-CNBHD interface, because G574 sits at the interface and both substitutions, despite introducing oppositely charged side chains, produce similar phenotypes”

New figure 2G and 2H

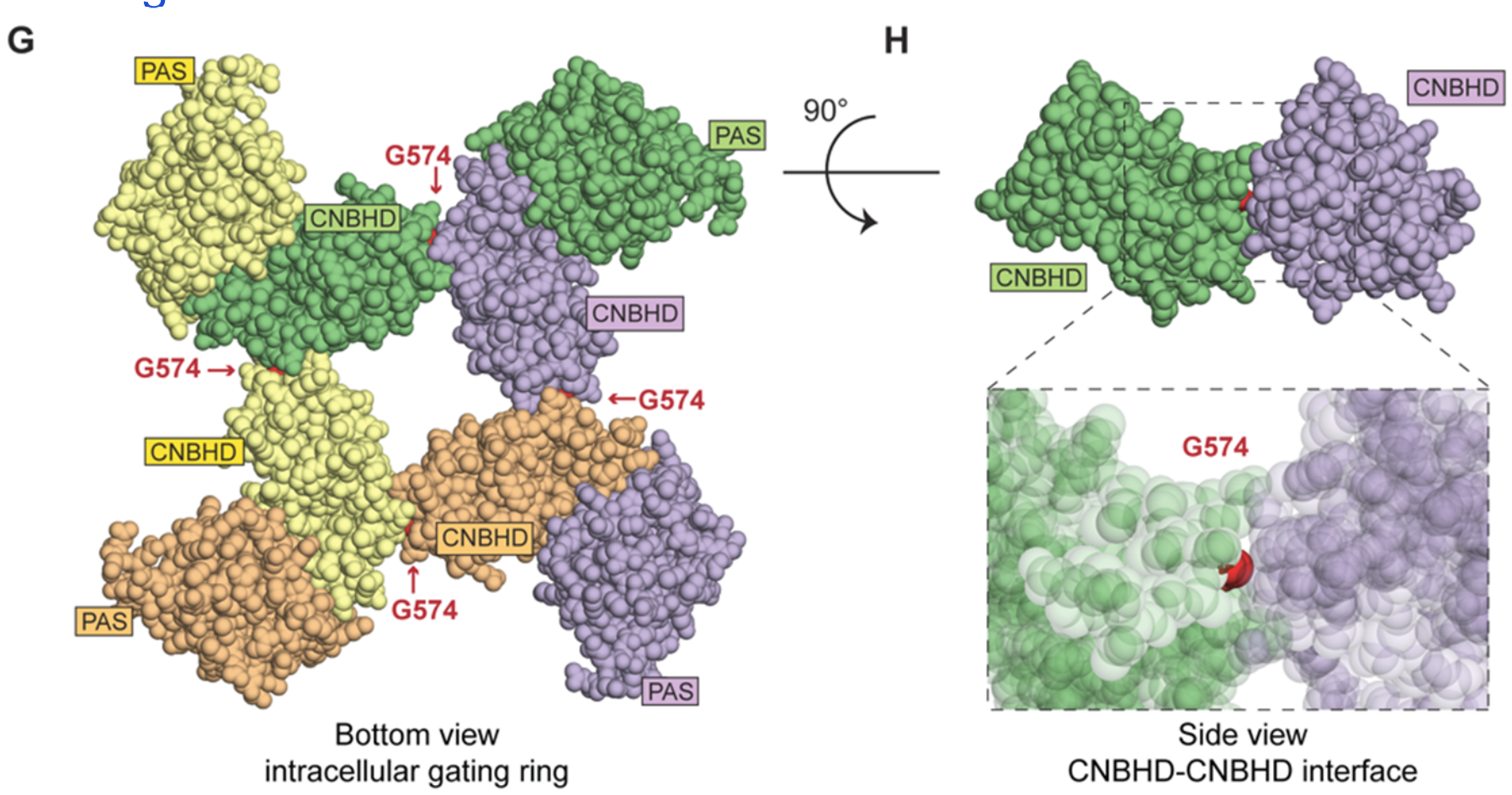

Figure legend of Figure 2G and 2H (Lines 827–838).

“(**G-H**) Sphere representations of the AlphaFold3-predicted structure of the homotetrameric EGL-2 channel, showing the inter-subunit arrangement of the PAS domains and CNBHDs within the intracellular ring. The four subunits are colored yellow, green, orange, and purple. Other domains are omitted for better visualization of G574 (in red) at the CNBHD-CNBHD interface. (G) Bottom view of the intracellular gating ring of EGL-2 channel. The CNBHDs form the inner ring, with the PAS domains positioned peripherally. (H) Side view of two CNBHDs from adjacent subunits, highlighting the position of G574 (in red) at the interface, generated by a 90° downward rotation of the bottom views shown in (G) and displayed at higher magnification, with the PAS domains omitted for clarity. Top, opaque sphere representation of two adjacent CNBHDs; bottom, magnified view of a smaller region containing the CNBHD-CNBHD interface at 70% transparency.”
