# Supplementary material for: Forward genetics reveals a conserved CNBHD-CNBHD interface that restrains EAG channel activation to regulate sleep
Source: PLoS Genet. 2026 Oct 1;22(10):e1012255. doi: 10.1371/journal.pgen.1012255 (PMC13660981; doi:10.1371/journal.pgen.1012255)
Supplement: Peer review history

# Peer review: Decision Letter 0

**DOI:** 10.1371/journal.pgen.1012255.r001

**Authors:** Andrew Chisholm, Fengwei Yu

18 Aug 2026

PGENETICS-D-26-00728

Forward genetics reveals a conserved CNBHD-CNBHD interface that restrains EAG channel activation to regulate sleep

PLOS Genetics

Dear Dr. Wang,

Thank you for submitting your manuscript to PLOS Genetics. The reviewers were generally enthusiastic about your paper but had a number of questions and comments. Therefore, we invite you to submit a revised version of the manuscript that addresses the points raised during the review process.  As you will see, these are mostly points of clarification and should not require additional experiments.

Please submit your revised manuscript within by Sep 17 2026 11:59PM. If you will need more time than this to complete your revisions, please reply to this message or contact the journal office at plosgenetics@plos.org. When you're ready to submit your revision, log on to https://www.editorialmanager.com/pgenetics/ and select the 'Submissions Needing Revision' folder to locate your manuscript file.

Please include the following items when submitting your revised manuscript:

* A letter that responds to each point raised by the editor and reviewer(s). You should upload this letter as a separate file labeled 'Response to Reviewers'. This file does not need to include responses to formatting updates and technical items listed in the 'Journal Requirements' section below.

* A marked-up copy of your manuscript that highlights changes made to the original version. You should upload this as a separate file labeled 'Revised Manuscript with Track Changes'.

* An unmarked version of your revised paper without tracked changes. You should upload this as a separate file labeled 'Manuscript'.

If you would like to make changes to your financial disclosure, competing interests statement, or data availability statement, please make these updates within the submission form at the time of resubmission. Guidelines for resubmitting your figure files are available below the reviewer comments at the end of this letter.

As the corresponding author, your ORCID iD is verified in the submission system and will appear in the published article. PLOS supports the use of ORCID, and we encourage all coauthors to register for an ORCID iD and use it as well. Please encourage your coauthors to verify their ORCID iD within the submission system before final acceptance, as unverified ORCID iDs will not appear in the published article. Only the individual author can complete the verification step; PLOS staff cannot verify ORCID iDs on behalf of authors.

We look forward to receiving your revised manuscript.

Kind regards,

Andrew D. Chisholm

Academic Editor

PLOS Genetics

Fengwei Yu

Section Editor

PLOS Genetics

Aimée Dudley

Editor-in-Chief

PLOS Genetics

Anne Goriely

Editor-in-Chief

PLOS Genetics

**Additional Editor Comments (if provided):**

**Journal Requirements:**

If the reviewer comments include a recommendation to cite specific previously published works, please review and evaluate these publications to determine whether they are relevant and should be cited. There is no requirement to cite these works unless the editor has indicated otherwise.

1) Please ensure that the CRediT author contributions listed for every co-author are completed accurately and in full.

At this stage, the following Authors/Authors require contributions: Xinyu Huang, Sudharsan Kannan, Gail A. Robertson, and Han Wang. Please ensure that the full contributions of each author are acknowledged in the "Add/Edit/Remove Authors" section of our submission form.

The list of CRediT author contributions may be found here: https://journals.plos.org/plosgenetics/s/authorship#loc-author-contributions

2) We do not publish any copyright or trademark symbols that usually accompany proprietary names, eg ©,  ®, or TM  (e.g. next to drug or reagent names). Therefore please remove all instances of trademark/copyright symbols throughout the text, including:

- TM on pages: 22, and 28.

**Reviewers' comments:**

Reviewer's Responses to Questions

**Comments to the Authors:**

**Please note here if the review is uploaded as an attachment.**

Reviewer #1: Starting from a forward genetics screen for sleep mutants in C. elegans Huang and colleagues describe the effect of a conserved glycine mutation (G574E) In the EGL-2 potassium channel on sleep in C. elegans. They use a combination of forward discovery genetics, in vivo and in oocytes structure-function analysis, cell specific rescue experiments, and calcium imaging in live behaving animals. They make use of Xenopus oocyte electrophysiology to perform 2 electrode voltage clamp experiments in order to characterize the biophysical changes conferred by the channel mutation. They conclude that the G574E mutation results in a “gain of function” effect both in vivo and in cellulo. The effect of the mutated channel maps to the ALA neuron.

This paper would be of interest to sleep researchers, ion channel physiologists, behavioral geneticists, and human geneticist who study potassium channelopathies.

The experiments are technically sound, the writing is clear, and the data are presented logically. I have only minor comments.

1. The authors make a strong case for G574E being a gain-of-function allele of egl-2. However, in figure 1, they show that introducing a wild-type copy of egl-2 into the egl-3(G574E) mutant fully rescues the sleep defect. This does not bother me (as I can imagine that the excess wild-type copies in the transgene array could compete away the channels with elevated potassium conductance), but should be commented on

2. They provide evidence that G574E is acting specifically in the ALA sleep-promoting neuron to reduce its excitability. But since egl-2 is expressed widely, I am surprised the mutant does not show more pleiotropic phenotypes such as locomotion, egg laying, pharyngeal pumping, and defecation defects. The authors should comment on this apparent lack of pleiotropy.

3. Were the G574E mutants defective in sleep induced by other stressors? E.g. UVC and Cry5B.

4. The authors propose that their results show a role for potassium channels in sleep. But, they only saw phenotypes with the gof mutant. The lof had no apparent phenotype in their experiments. Hence, it is unclear to what degree EGL-2 wt protein (i.e. one that is not mutated) is involved in normal sleep regulation.

5. The authors use the nlp-67 promoter in order to express transgenes specifically in the ALA neuron. Can they provide data or a reference that shows this specificity?

6. I would like to see more details on their method for heat shocking as this assay is notoriously noisy/variable. Specifically, did the worms have access to ample food during the heat shock? Were the plates immersed in the water bath right-side up or upside down? What was the volume of NGM agar on the plate? How old were the NGM plates?

7. The authors use the term “standard dissecting microscope”. Is the word “standard” necessary?

8. Why was imaging egl-2 in the ALA neuron performed in the L4 stage? Why not in adults?

Reviewer #2: In the manuscript, “Forward genetics reveals a conserved CNBHD-CNBHD interface that restrains EAG channel activation to regulate sleep”, the authors identified a function for the cyclic nucleotide binding homology domain in the C. elegans-encoded EGL-2 voltage-gated K+ channel. The authors used the mutagen EMS to induce point mutations into the C. elegans genome, and then they conducted a nice forward genetics screen to identify a mutation that interfered with the ALA mechanosensory interneuron’s function to promote behavioral quiescence after heat-shock induction of LIN-3/EGF.

The authors found that an EMS-induced mutation changed a conserved glycine to a glutamate amino acid in the cyclic nucleotide binding domain of EGL-2, causing the channel to show phenotypic properties reminiscent of unregulated K+ conduction. The experiments presented in the results sections convincingly demonstrate that the mutant gain-of-function (gf) allele promotes premature and/or excessive EGL-2 -mediated K+ conduction, which attenuates depolarization of the ALA, and consequently interferes with heat-shock LIN-3 expression- or experimental heat stressed-induced behavioral quiescence.

The authors further explored the function of EGL-2’s CNBHD region by introducing additional missense mutations in the CNBHD and the intrinsic ligand domain. From their well-controlled experiments, they conclude that interactions between CNBHD and the intrinsic ligand domain regulate the activation and deactivation kinetics of ion conduction.

Finally, the authors demonstrated that their findings apply to other voltage-gated KCNH channels. They introduced the CNBHD gain-of-function mutation into the C. elegans ERG homolog UNC-103 and in the human K+ channel hERG1a. From an egg-laying behavioral assay, the authors showed that mutant UNC-103, containing the CNBHD (gf) allele, reduced the frequency of egg-laying muscle contractions. Additionally, from electrophysiological studies using xenopus oocytes, the authors showed that mutant hERG, containing the CNBHD (gf) allele, display accelerated K+ conductance at more negative voltages.

The experiments, detailed in the manuscript, were well-executed and conservatively interpreted. The identification of the gain-of-function allele underscores the utility of using chemical-mediated random mutagenesis and forward genetic screens to uncover functions for distinct domains in biomolecules; the conclusions from the experiments in this manuscript provide an important understanding on how a specific region at the interface of the intracellular cyclic nucleotide binding domains between KCNH subunits regulate the gating of ion conductance through the S5 and S6 transmembrane helixes. The manuscript was an easy read for me, and I do not have any significant issues with the research presented in the paper.

Signed, L. Rene Garcia

Minor comments:

1. In the abstract, page 2 line 33-34. The sentence, “In vivo calcium imaging showed that the gain-of-function mutation inhibits ALA neuronal activity to suppress sleep, consistent…”, can be misread by the casual reader as if the ALA normal function is to suppress sleep, and the (gf) mutation inhibits that function. A clearer way to state this might be something like, “In vivo calcium imaging showed that the gain-of-function mutation suppresses sleep by inhibiting ALA neuronal activity, consistent with enhanced potassium channel function.

2. In the Discussion, under the section: EAG channels and sleep regulation, one should comment in one or two sentences why in Figure 4, panel B through D, there was no obvious abnormal phenotype for reducing or eliminating EGL-2 activity on ALA’s regulation of behavioral quiescence; one can mention other potential compensating K+ channels in ALA that can substitute for EGL-2 normal function.

Reviewer #3: This manuscript reports a forward genetic screen in C. elegans to identify genes involved in sleep and identified a gain of function (GOF) mutation G574E in a KCNH potassium channel, EAG (EGL-2 in C. Elegans) which is responsible for a loss of sleep, which is an interesting and novel result. G574E is located in the C-terminal CNBH Domain, is a conserved residue, and is nearby the same residue in an adjacent CNBH Domain from an adjacent subunit.

Pros

Elegantly done studies to reintroduce WT EGL-2 to mutant G574E worms and perform a Crispr introduction of G574E to WT worms to confirm the behavioral phenotype and link to G574E.

Additionally, re-introduction of EGL-2 / EAG channels with engineered point mutations that block the K ion flux out of the cell (or into the cell) indicated the EAG mutant channel effect was due to the movement of K ions.

Convincing data to show activity of the ALA sleep promoting neurons was impaired by EGL-2 G574E, consistent with GOF of a K channel, presumably due to increased membrane hyperpolarization.

To expand the scope of this finding, they also generate C. elegans in with the homologous EAG mutant in the unc103 worm (G427R), which is the locus of ERG (G749R), a channel in the same KCNH family as EAG. The unc103/ERG mutant worms have a gain of function phenotype (egg retention) in their egg laying behavior which generalizes the importance of the G574E mutant to other channels and behaviors.

The proposed function of EAG mutants as inferred by behavioral and cellular experiments is examined directly in ERG channels with the equivalent hERG G749R mutant in heterologous expression experiments. hERG G749R is shown here to have altered gating, including gain of function in some functional properties (increase in the current density and left shift of the gV). Faster deactivation in these channels, however, could be considered a LOF. All the gating effects are consistent with a disruption of the CNBHD and allosteric effects on the gating machinery.

Gain of function in channel behavior measured directly in ERG G749R is consistent with the egg laying retention and, by extension, consistent with a GOF in EGL-2 G574E, b/c a GOF would hyperpolarize and decrease ALA neuronal activity and decrease activity in the sleep promoting ALA neurons and loss of sleep.

Clarifications

Please clarify on the rationale and justification for the experiment with G574E and intrinsic ligand YNL to ANA mutants, perhaps in the Results or Discussion. Was the idea that the ANA mutant would right-shift the voltage dependence and offset the presumed left-shifted voltage dependence of G574E? The conclusion that the intact YNL is necessary for the sleep phenotype might only be true in the context of G574E (and not for instance in G574R if it has a greater GOF than G574E).

Other than the nearness at G574 in the structures and models, there is not much buried surface between CNBHDs and not much evidence that the CNBHDs of these channels interact directly. Perhaps include distance markers in the figures between the G574 and G574 to show more clearly. A change in gating with a mutant does not necessarily mean that a CNBHD-CNBHD interaction is disrupted. This result could also be consistent with a change in individual CNBHD dynamics or individual CNBHD communication with the pore. I would suggest the authors soften that conclusion a bit throughout the article.

**********

**Have all data underlying the figures and results presented in the manuscript been provided?**

Large-scale datasets should be made available via a public repository as described in the *PLOS Genetics* data availability policy, and numerical data that underlies graphs or summary statistics should be provided in spreadsheet form as supporting information.

Reviewer #1: Yes

Reviewer #2: Yes

Reviewer #3: Yes

**********

PLOS authors have the option to publish the peer review history of their article (what does this mean?). If published, this will include your full peer review and any attached files.

If you choose “no”, your identity will remain anonymous but your review may still be made public.

**Do you want your identity to be public for this peer review?** For information about this choice, including consent withdrawal, please see our Privacy Policy.

Reviewer #1: No

Reviewer #2: **Yes:**  L Rene Garcia

Reviewer #3: No

[NOTE: If reviewer comments were submitted as an attachment file, they will be attached to this email and accessible via the submission site. Please log into your account, locate the manuscript record, and check for the action link "View Attachments". If this link does not appear, there are no attachment files.]

**Figure resubmission:**

While revising your submission, we strongly recommend that you use PLOS’s NAAS tool (https://ngplosjournals.pagemajik.ai/artanalysis) to test your figure files. NAAS can convert your figure files to the TIFF file type and meet basic requirements (such as print size, resolution), or provide you with a report on issues that do not meet our requirements and that NAAS cannot fix.

After uploading your figures to PLOS’s NAAS tool - https://ngplosjournals.pagemajik.ai/artanalysis, NAAS will process the files provided and display the results in the "Uploaded Files" section of the page as the processing is complete. If the uploaded figures meet our requirements (or NAAS is able to fix the files to meet our requirements), the figure will be marked as "fixed" above. If NAAS is unable to fix the files, a red "failed" label will appear above. When NAAS has confirmed that the figure files meet our requirements, please download the file via the download option, and include these NAAS processed figure files when submitting your revised manuscript.

**Reproducibility:**

To enhance the reproducibility of your results, we recommend that authors deposit laboratory protocols in protocols.io, where a protocol can be assigned its own identifier (DOI) such that it can be cited independently in the future. Additionally, PLOS ONE offers an option to publish peer-reviewed clinical study protocols. Read more information on sharing protocols at https://plos.org/protocols?utm_medium=editorial-email&utm_source=authorletters&utm_campaign=protocols

# Author comment: Author response to Decision Letter 1

**DOI:** 10.1371/journal.pgen.1012255.r002

15 Sep 2026

Attachment Submitted filename: response to reviewer comment egl-2 v4 final.pdf

# Editor report: Decision Letter 1

**DOI:** 10.1371/journal.pgen.1012255.r003

**Authors:** Andrew Chisholm, Fengwei Yu

17 Sep 2026

Dear Dr Wang,

We are pleased to inform you that your manuscript entitled "Forward genetics reveals a conserved CNBHD-CNBHD interface that restrains EAG channel activation to regulate sleep" has been editorially accepted for publication in PLOS Genetics. Congratulations!

Before your submission can be formally accepted and sent to production you will need to complete our formatting changes, which you will receive in a follow up email. Please be aware that it may take several days for you to receive this email; during this time no action is required by you. Please note: the accept date on your published article will reflect the date of this provisional acceptance, but your manuscript will not be scheduled for publication until the required changes have been made.

Once your paper is formally accepted, an uncorrected proof of your manuscript will be published online ahead of the final version, unless you’ve already opted out via the online submission form. If, for any reason, you do not want an earlier version of your manuscript published online or are unsure if you have already indicated as such, please let the journal staff know immediately at plosgenetics@plos.org.

In the meantime, please log into Editorial Manager at https://www.editorialmanager.com/pgenetics/, click the "Update My Information" link at the top of the page, and update your user information to ensure an efficient production and billing process. Note that PLOS requires an ORCID iD for all corresponding authors. Therefore, please ensure that you have an ORCID iD and that it is validated in Editorial Manager. To do this, go to ‘Update my Information’ (in the upper left-hand corner of the main menu), and click on the Fetch/Validate link next to the ORCID field.  This will take you to the ORCID site and allow you to create a new iD or authenticate a pre-existing iD in Editorial Manager.

If you have a press-related query, or would like to know about making your underlying data available (as you will be aware, this is required for publication), please see the end of this email. If your institution or institutions have a press office, please notify them about your upcoming article at this point, to enable them to help maximise its impact. Inform journal staff as soon as possible if you are preparing a press release for your article and need a publication date.

Thank you again for supporting open-access publishing; we are looking forward to publishing your work in PLOS Genetics!

Yours sincerely,

Andrew D. Chisholm

Academic Editor

PLOS Genetics

Fengwei Yu

Section Editor

PLOS Genetics

Aimée Dudley

Editor-in-Chief

PLOS Genetics

Anne Goriely

Editor-in-Chief

PLOS Genetics

www.plosgenetics.org

BlueSky: @plos.bsky.social

----------------------------------------------------

Comments from the reviewers (if applicable):

----------------------------------------------------

**Data Deposition**

If you have submitted a Research Article or Front Matter that has associated data that are not suitable for deposition in a subject-specific public repository (such as GenBank or ArrayExpress), one way to make that data available is to deposit it in the Dryad Digital Repository. As you may recall, we ask all authors to agree to make data available; this is one way to achieve that. A full list of recommended repositories can be found on our website.

The following link will take you to the Dryad record for your article, so you won't have to re‐enter its bibliographic information, and can upload your files directly:

http://datadryad.org/submit?journalID=pgenetics&manu=PGENETICS-D-26-00728R1

More information about depositing data in Dryad is available at http://www.datadryad.org/depositing. If you experience any difficulties in submitting your data, please contact help@datadryad.org for support.

Additionally, please be aware that our data availability policy requires that all numerical data underlying display items are included with the submission, and you will need to provide this before we can formally accept your manuscript, if not already present.

----------------------------------------------------

**Press Queries**

If you or your institution will be preparing press materials for this manuscript, or if you need to know your paper's publication date for media purposes, please inform the journal staff as soon as possible so that your submission can be scheduled accordingly. Your manuscript will remain under a strict press embargo until the publication date and time. This means an early version of your manuscript will not be published ahead of your final version. PLOS Genetics may also choose to issue a press release for your article. If there's anything the journal should know or you'd like more information, please get in touch via plosgenetics@plos.org.

# Editor report: Acceptance letter

**DOI:** 10.1371/journal.pgen.1012255.r004

**Authors:** Andrew Chisholm, Fengwei Yu

PGENETICS-D-26-00728R1

Forward genetics reveals a conserved CNBHD-CNBHD interface that restrains EAG channel activation to regulate sleep

Dear Dr Wang,

We are pleased to inform you that your manuscript entitled "Forward genetics reveals a conserved CNBHD-CNBHD interface that restrains EAG channel activation to regulate sleep" has been formally accepted for publication in PLOS Genetics! Your manuscript is now with our production department and you will be notified of the publication date in due course.

The corresponding author will soon be receiving a typeset proof for review, to ensure errors have not been introduced during production. Please review the PDF proof of your manuscript carefully, as this is the last chance to correct any errors. Please note that major changes, or those which affect the scientific understanding of the work, will likely cause delays to the publication date of your manuscript.

Soon after your final files are uploaded, unless you have opted out or your manuscript is a front-matter piece, the early version of your manuscript will be published online. The date of the early version will be your article's publication date. The final article will be published to the same URL, and all versions of the paper will be accessible to readers.

For Research Articles, you will receive an invoice from PLOS for your publication fee after your manuscript has reached the completed accept phase. If you receive an email requesting payment before acceptance or for any other service, this may be a phishing scheme. Learn how to identify phishing emails and protect your accounts at https://explore.plos.org/phishing.

Thank you again for supporting PLOS Genetics and open-access publishing. We are looking forward to publishing your work!

With kind regards,

Sharmila Kamatchi

PLOS Genetics

On behalf of:

The PLOS Genetics Team

Carlyle House, Carlyle Road, Cambridge CB4 3DN | United Kingdom

plosgenetics@plos.org | +44 (0) 1223-442823

plosgenetics.org | Twitter: @PLOSGenetics
